# Long range personalized cancer treatment strategies incorporating evolutionary dynamics

**DOI:** 10.1186/s13062-016-0153-2

**Published:** 2016-10-22

**Authors:** Chen-Hsiang Yeang, Robert A. Beckman

**Affiliations:** 1 Institute of Statistical Science, Academia Sinica, Taipei, Taiwan; 2 Departments of Oncology and of Biostatistics, Bioinformatics, and Biomathematics, Lombardi Comprehensive Cancer Center and Innovation Center for Biomedical Informatics, Georgetown University Medical Center, Washington, DC USA

**Keywords:** Dynamic precision medicine, Evolution, Intratumoral heterogeneity, Precision medicine, Treatment strategies, Dynamic programming

## Abstract

**Background:**

Current cancer precision medicine strategies match therapies to static consensus molecular properties of an individual’s cancer, thus determining the next therapeutic maneuver. These strategies typically maintain a constant treatment while the cancer is not worsening. However, cancers feature complicated sub-clonal structure and dynamic evolution. We have recently shown, in a comprehensive simulation of two non-cross resistant therapies across a broad parameter space representing realistic tumors, that substantial improvement in cure rates and median survival can be obtained utilizing dynamic precision medicine strategies. These dynamic strategies explicitly consider intratumoral heterogeneity and evolutionary dynamics, including predicted future drug resistance states, and reevaluate optimal therapy every 45 days. However, the optimization is performed in single 45 day steps (“single-step optimization”).

**Results:**

Herein we evaluate analogous strategies that think multiple therapeutic maneuvers ahead, considering potential outcomes at 5 steps ahead (“multi-step optimization”) or 40 steps ahead (“adaptive long term optimization (ALTO)”) when recommending the optimal therapy in each 45 day block, in simulations involving both 2 and 3 non-cross resistant therapies. We also evaluate an ALTO approach for situations where simultaneous combination therapy is not feasible (“Adaptive long term optimization: serial monotherapy only (ALTO-SMO)”). Simulations utilize populations of 764,000 and 1,700,000 virtual patients for 2 and 3 drug cases, respectively. Each virtual patient represents a unique clinical presentation including sizes of major and minor tumor subclones, growth rates, evolution rates, and drug sensitivities.

While multi-step optimization and ALTO provide no significant average survival benefit, cure rates are significantly increased by ALTO. Furthermore, in the subset of individual virtual patients demonstrating clinically significant difference in outcome between approaches, by far the majority show an advantage of multi-step or ALTO over single-step optimization. ALTO-SMO delivers cure rates superior or equal to those of single- or multi-step optimization, in 2 and 3 drug cases respectively.

**Conclusion:**

In selected virtual patients incurable by dynamic precision medicine using single-step optimization, analogous strategies that “think ahead” can deliver long-term survival and cure without any disadvantage for non-responders. When therapies require dose reduction in combination (due to toxicity), optimal strategies feature complex patterns involving rapidly interleaved pulses of combinations and high dose monotherapy.

**Reviewers:**

This article was reviewed by Wendy Cornell, Marek Kimmel, and Andrzej Swierniak. Wendy Cornell and Andrzej Swierniak are external reviewers (not members of the Biology Direct editorial board). Andrzej Swierniak was nominated by Marek Kimmel.

**Electronic supplementary material:**

The online version of this article (doi:10.1186/s13062-016-0153-2) contains supplementary material, which is available to authorized users.

## Background

A major trend in molecular oncology is the development of targeted therapies tailored to particular molecular abnormalities. Tumors are stratified by molecular characteristics and matched to appropriate therapies. Personalization has the potential to increase the percentage of patients who benefit from therapy, thus increasing the average benefit and decreasing the cost of development [1]. However, personalization is driven by the average or consensus molecular characteristics of a tumor cell mixture, and by a static picture of the current molecular state. In addition, current approaches focus primarily on optimizing the next therapeutic maneuver. Recently, simulations have shown that dynamic precision medicine treatment paradigms which take into account sub-clonal diversity within individual tumors and their dynamic evolution may produce superior clinical outcomes [2]. Whereas static personalized therapy has the potential for short term benefit, and relapse is common, dynamic precision medicine yields a significantly higher cure rate. While these approaches are more forward looking in that they consider future risks, optimization still focuses on the next therapeutic maneuver.

Genetic instability has been postulated to be fundamental to tumor evolution [3]. Mathematical models have demonstrated that early acquisition of genetic instability increases the efficiency of carcinogenesis, making it more probable that clinically apparent tumors will be genetically unstable [4–6]. These models predicted a high level of mutational burden and associated subclonal structure in tumors. Moreover, the parallel evolution of multiple subclones featured in the models led to an explicit prediction of divergent and convergent evolution as an expected feature of tumors, insofar as it was stated that different subclones would have partially overlapping sets of driver genes or alterations [7]. These predictions were subsequently verified by seminal experimental observations including sub-clonal structure and phylogenetic evolution in leukemias [8–10], divergence between the molecular characteristics of primary and metastatic lesions in solid tumors [11, 12], molecular variation and convergent evolution within different spatial locations within a single renal cell cancer lesion [13], and a high burden of approximately 20,000-30,000 mutations per solid tumor [14], including approximately 50-100 non-synonymous clonal mutations within exons [15, 16]. Even greater diversity would presumably be revealed by deeper sequencing [17].

The molecular diversity and genetic instability imply the possibility of pre-existing and acquired drug resistance, respectively, that can be selected for by therapy [18]. For example, multiple mechanisms of heritable (“heritable” herein refers to stable genetic and or epigenetic changes of somatic cells, which are passed on to daughter cells) resistance have been documented for erlotinib and gefitinib [19, 20], and the sensitive sub-clone may also persist, leading to rebound if therapy is withdrawn [21]. Similarly, a variety of resistance pathways are known for crizotinib, and more than one can co-exist within the same patient [22, 23]. In chronic myeloid leukemia (CML), resistance is generally due to mutations in the single fusion gene that drives the malignancy, and combinations may be useful to delay the emergence of multiple resistance [24, 25].

Within a single heritable state, non-genetic mechanisms of resistance are already hard-wired within signaling networks. Examples include feedback resistance to vemurafenib in BRAF mutant colorectal cancer [26, 27], and feedback upregulation of tyrosine kinase receptors in response to therapy with phosphatidyl inositol-3-kinase inhibitors [28]. Because of these rapid non-heritable resistance mechanisms, combination therapy may be required merely to effectively treat a single genetic or epigenetic heritable somatic state.

Current precision medicine for cancer matches the consensus molecular pattern of a tumor to single agent or combination therapy, and continues treatment until tumor worsening or relapse. At that time, the process of evaluating the tumor’s molecular characteristics and matching to a new therapy is repeated. This approach represents a great advance over previous non-selective approaches. Yet the complicated dynamics of resistance suggests the additional need for direct, explicit consideration of intratumoral heterogeneity and dynamics.

We have developed methods for comprehensive comparisons of complex cancer treatment strategies for metastatic cancer. A strategy is not a specific treatment sequence, but rather a data-driven method for planning treatment sequences. A strategy may suggest which therapies to utilize at treatment initiation, when to switch therapies, when to use high dose monotherapy, and when to use combinations. Strategies individualize treatment sequences based on both static and dynamic features. The current dominant precision medicine paradigm is an example of a strategy, but it is not the only possible one.

In previous work, we compared the current personalized medicine strategy to five other dynamic precision medicine strategies that considered sub-clonal heterogeneity and evolutionary dynamics and predicted future states 45 days after treatment initiation, in the setting of metastatic cancer [2]. A situation was simulated in which two non-cross resistant drugs are available for treatment. These non-cross resistant drugs are intended for different subclones with different drivers and/or alterations, and may work on different pathways. (Operationally, we define “non-cross resistant” drugs as drugs for which no single molecular alteration is known which will simultaneously confer resistance to both agents. Knowledge of presence or absence of cross resistance may come from in vitro forward mutation assays or from clinical data. “Non cross resistant” does not simply mean that the drugs work by a different mechanism, as often drugs working by different mechanisms may still be subject to a common mechanism of resistance. For example, molecular alterations to the final common pathway for apoptosis may lead to resistance to a variety of agents, and upregulation of small molecule efflux transporters may also confer simultaneous resistance to multiple agents). Each “drug” may really be a combination if multiple drugs are needed to knock out a single pathway. However, we will subsequently refer to these single agents or combinations as “drug 1” and “drug 2”. Every 45 days, an optimal treatment was selected for the next 45 days, where available options were either full dose drug 1, full dose drug 2, or simultaneous combinations of both drugs at reduced dose due to enhanced toxicity associated with simultaneous administration (such a constraint is common but not universal for all drug combinations, but was chosen as the most realistic scenario for a generic simulation). Thus, treatment could be adaptively adjusted every 45 days. (A 45 day interval was chosen as a rounded number approximating every six weeks. Oncology patients typically receive therapy every 3 weeks with the break in between to allow the bone marrow and intestines to recover from typical toxicities, and are thus returning to the clinic every 3 weeks. In clinical trials, tumor burden is evaluated by computed tomography in intervals of every six weeks or more. Thus, approximately every six weeks was chosen as the interval for assessment to coincide with other clinical activities. The effect of the interval between adaptive treatment adjustments is an interesting area for future research). The optimal treatment for the next 45 days was selected in three stages. In the first stage, an evolutionary model was employed using patient input data (Methods) to estimate which treatment option would be predicted to give the fewest number of total surviving tumor cells (summed over all lesions) at the next 45 day time point. In the second stage, the evolutionary model was employed to estimate which treatment option would give the lowest probability of forming a single cell which was simultaneously resistant to both available therapies, and therefore “incurable”. The treatment recommendations emerging from stages 1 and 2 would typically differ. Hence, a third stage would be applied in which a “strategy”, ie a set of prespecified rules, would be applied to prioritize between the recommendations of the first two stages, leading to a final recommendation for the next 45 day treatment period.

The simplest dynamic strategy was strategy 1, in which the treatment was chosen for each treatment step that was predicted to minimize the number of cancer cells remaining at the end of the treatment step. Strategy 2.1 began to prioritize prevention of multiple resistance. The strategy selected the treatment that would minimize the risk of formation of doubly resistant cells, *unless the patient had disease visible on computed tomography* (represented in the simulation as 10^9^ or more cells), in which case the strategy selected treatment which would minimize the total cell number. The most successful strategy, however, was termed “strategy 2.2”, and it amounts to more aggressive prevention of multiple resistance than strategy 2.1. In essence, the strategy selects the treatment which minimizes the probability of forming a doubly resistant cell, *unless the patient is in imminent danger of death, severe injury, or severe discomfort from the total tumor burden*, in which case the treatment is selected which minimizes the tumor burden. In the simulation, each patient started with a burden of 10^9^ cells (a single 1 cm^3^ lesion), and only if the total cell number increased to 10^11^ or more cells would strategy 2.2 choose the treatment that focused on minimizing the total cell number. In real applications, the treating physician could apply her/his judgement about whether the patient was in immediate danger. The simulation also included strategy 0, the current precision medicine strategy, which was to apply the therapy which was most effective for the largest clone and to continue as long as the cancer was not worsening, repeating the process at that time. Two other dynamic strategies were evaluated: a complete listing of the strategies and definitions are given in Additional file 1: Table S1, Supplementary Results.

The simulations were performed over a large parameter space based on a broad survey of clinical experience and experimental literature, and represent a sensitivity analysis over the range of known realistic tumor states [29–38]. Each virtual patient represented a unique parameter set of net growth rates, drug sensitivities, initial sub-populations, and genetic/epigenetic transition rates between the heritable states. Three million parameter configurations were considered based on the literature survey and clinical experience, and the virtual patients represent a thorough sampling of possible oncology scenarios. The comprehensive sensitivity analysis over a very large number of virtual patients with characteristics based on experimental and clinical data differentiates this work and the current study from other efforts in this field, in which a small number of experimental data points are assumed to be generalizable when constructing models. Lack of generalizability is a major problem with both theoretical and experimental studies in oncology [7]. In this case, a variety of sources were used to ensure that the parameter ranges were realistic as well as sufficiently broad to encompass all likely oncology scenarios. These included preclinical and clinical literature as well as experience of one of us in oncology patient care and clinical research, comprising clinical studies of more than two dozen experimental oncology agents in most major tumor types and thousands of patients over several decades. Details of parameter selection are given in Methods.

Dynamic precision medicine strategies dramatically improved patient outcomes compared to the current personalized medicine strategy. Mean and median survival times were doubled and cure rates improved from less than 1 % to 15-20 %. For comparison, a new therapeutic agent is generally approvable for marketing by national health authorities if it improves survival times by 25 % with or without increased cure rates. The results were driven by 1/3 of the virtual patients who had substantial benefits from dynamic precision medicine strategies. The patients who benefited spanned most of possible tumor and drug characteristics, except they all had some level of pre-existing heterogeneity or genetic instability. In comparison, a new therapeutic agent is typically limited to one or a small number of clinical scenarios. Thus, the enormous current efforts directed at discovery and development of new agents might be complemented by efforts to use these agents optimally.

Some strategies were more effective than others based on underlying dynamics. Thus both therapies and the strategies used for planning them may require individualization. The current personalized medicine strategy was not optimal for any of the 3 million virtual patients, representing a comprehensive survey of tumor and therapy characteristics.

We have pointed out that complicated dynamics also calls for long range planning, not merely single step reactive measures, drawing an analogy to optimal chess play [39, 40]. In a comprehensive editorial, the editor-in-chief of *Nature Reviews Clinical Oncology* independently echoed our analogy to chess: “World class players win by thinking at least 15 steps ahead of their next move, and by predicting their opponent’s tactics well in advance. It seems that what we are doing in the fight against cancer is more a one-step reactive approach to its next move. No wonder we feel like we are losing this game!” [41]. Herein we comprehensively evaluate the benefits of long range planning, by directly simulating thinking 5-40 steps ahead.

**Fig. 1:**
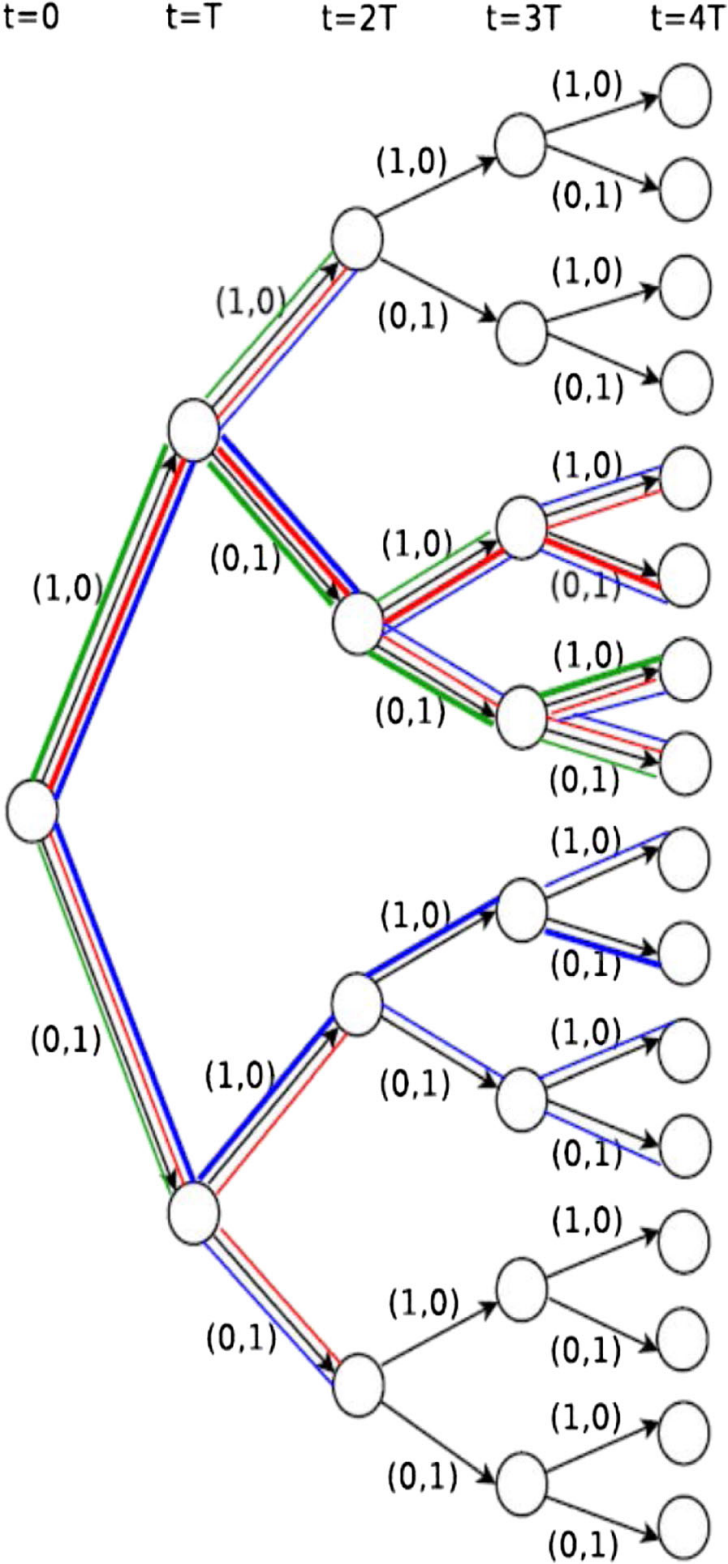
Example of single-step heuristics, multi-step heuristics, and global optimization on a decision tree. The example tree is spanned by 4-step treatment sequences with only two possible dosage combinations (full drug 1 dosage (1*,* 0) and full drug 2 dosage (0*,* 1)) in each step. Each node represents a population structure and the two edges emitting from each internal node represent the two possible treatments following the observed population structure. A path represents a treatment sequence. The treatment sequences traversed by three strategies are marked with distinct colors: green – one-step heuristic, red – two-step heuristic, blue – global optimization algorithm. The paths with bold colored lines are the treatment sequences selected by the strategies. A one-step heuristic chooses one of the two branching edges in each time step. A two-step heuristic chooses one of the four branching paths of length 2 in every two steps. Global optimization keeps the two best paths of length 2 at step 2, tracks the subsequent 4 paths for each earlier path and chooses the optimal one

While our previous work hinted at the value of thinking ahead, in that therapeutic maneuvers that prevented future resistance were often the optimal choice even when they did not provide optimal immediate reduction of tumor burden, optimization within each 45 day interval was based on projected outcomes at the end of that single interval, termed *single-step optimization, single-step strategies, or single-step heuristics* (Fig. 1). The term “heuristic” refers to a strategy which in part employs qualitative reasoning or clinical intuition, as in strategy 2.2 which considers the clinically familiar concepts of reduction of total disease burden and prevention of drug resistance. In this work, we consider the same strategies, but we use outcomes 5 intervals in the future to determine the optimal therapy for the each 45 day interval as the first step of a hypothetical 5 interval sequence, which we term *multi-step optimization, multi-step strategies, or multi-step heuristics* (Fig. 1)*.* The 5 step treatment sequence may in principle be reevaluated based on clinical results after step 1 if they diverge from predicted results. Finally, we considered *adaptive long-term optimization (ALTO)*, which examines potential outcomes 40 steps ahead in 45-day increments (a 5 year time span, or the length of the simulation) in determining each 45 day treatment selection as the first step in a 40 step sequence (Fig. 1). The decision tree of possible 40 step treatment sequences required for this exercise is very large, and unpromising branches must be pruned (see Methods). For ALTO, it is not computationally feasible to directly compute either overall survival or the probability of forming a multiply resistant cell over the large number of pathways in the decision tree even after pruning. Thus instead of the heuristic strategies from [2], we utilize a mathematical approximation to survival to more rapidly rank the overall treatment sequences (see Methods). In the main text, we focus primarily on single-step and multi-step versions of strategy 2.2, and ALTO, in addition to “adaptive long term optimization: serial monotherapy only (ALTO-SMO)” which applies the restrictive assumption that simultaneous combinations are impossible, in order to determine the effectiveness of monotherapy sequences if they are applied with frequent adaptation. A description of the other strategies, and data on their single-step and multi-step versions, is given in Additional file 1: Supplementary Results.

Finally, we also scale up to three drugs/combinations. Given the likely diversity in a population of tumor cells, a minimum of three non-cross resistant therapies (each single agents or combinations) will likely be required for cure. The model includes 8 heritable states (a 2 × 2 × 2 table of sensitivity versus resistance), and a higher dimensional parameter space. Highly parallel computing and a more focused parameter search are needed to span possible drug and tumor characteristics.

Our results demonstrate that long range planning offers highly significant advantages to selected patients, without any disadvantage for the others.

## Results

In this section, we will report on two results:

1. The effectiveness of multi-step heuristics 2.2 and ALTO when compared with single-step heuristics 2.2 and the current personalized medicine strategy, strategy 0. We will also compare ALTO-SMO to single-step heuristics which allows combinations. Note that because the 3 drug simulation contains more states and allows doubly resistant cells at time zero, results from it cannot be directly compared to results from the two drug simulation.
2. Examples of strategies from multi-step heuristic 2.2 and ALTO which achieved cures in patients who were incurable by single step heuristic 2.2.

### Effectiveness of multi-step heuristics and ALTO when compared with single-step heuristics and current personalized medicine

Table 1 shows the performance of single-step and multi-step versions of strategy 2.2, as well as that of ALTO, ALTO-SMO, and the current precision medicine strategy 0 (in strategy 0 the patient is treated with the best therapy for the largest subclone and this treatment is maintained until the tumor relapses or worsens, whereupon the process is repeated). The metrics reflect statistics across a population of approximately 760,000 virtual patients in the 2 drug case and 1.7 million virtual patients in the 3 drug case. Each virtual patient represents a unique set of parameters including number of cells for each type of subclone, growth rates, phenotypic transition rates between drug sensitivity and resistance, and drug sensitivity properties of these states. Like the population of virtual patients in [2], the population represents a comprehensive pan-oncology survey of reasonable parameter values, with the exception that the simulations in this work are restricted to “curable” patients for whom the available drugs have the ability to produce a negative growth rate in sensitive cells. In contrast, in [2] the drugs were required only to be able to slow the growth of the tumor by 25 % or more. In [2], continuous variation of the dose of drugs 1 and 2 was permitted, when they were given in simultaneous combination, subject to a cap on the sum of the doses equal to the permitted monotherapy dose. In this work, only a single dosing paradigm is allowed for simultaneous combination: half-dose of each drug for binary combinations, and one-third dose for triple combinations. The 2 drug simulation contains sensitive and resistance states to drug 1 and 2, amounting to a 2 × 2 table of phenotypic states, but doubly resistant states are not permitted at time zero. The 3 drug simulation contains a 2 × 2 × 2 table of phenotypic states corresponding to sensitivity and resistance to drugs 1, 2, and 3, and triply resistant states are not permitted at time zero. Additional details of the simulations are given below in conjunction with examples and in methods and Additional file 1: Supplementary methods.

**Table 1:** Comparison of treatment outcomes for 5 strategies

Strategy	Median 2	Median 3	5 yr 2	5 yr 3	Cure 2	Cure 3
0: Current personalized medicine	585	720	35.8	29.6	23.0	10.8
Single-step strategy 2.2	1170	1080	47.3	40.5	30.1	17.6
Multistep strategy 2.2	1215	1080	47.5	41.5	30.1	18.5
ALTO-SMO	855	1035	44.7	41.8	34.3	17.9
ALTO	1260	1080	47.5	43.2	36.8	25.4

Strategy 0 is the current personalized medicine strategy: treat with the best drug for the largest clone and continue to treat until tumor worsening or relapse, then rebiopsy and repeat. Strategy 2.2: select/adapt treatment every 45 days using evolutionary dynamic model to minimize the likelihood of forming a cell simultaneously resistant to all the therapies at a future reference timepoint, unless the estimated tumor burden is 10^11^ cells or more. Single-step strategy 2.2: future reference timepoint for selecting treatments is 45 days, corresponding to the interval between treatment adaptations. Multistep strategy 2.2: future reference timepoint for selecting treatments is 225 days, or 5 times the interval between treatment adaptations (“thinking 5 steps ahead”). ALTO: Adaptive long-term optimization. ALTO-SMO: Adaptive long term optimization-serial monotherapy only. Median 2: median survival days for two-drug cases. Median 3: median survival days for three-drug cases. 5 yr 2: percentage of two-drug cases with more than 5-year survival time. 5 yr 3: percentage of three-drug cases with more than 5-year survival time. Cure 2: percentage of two-drug cured cases. Cure 3: percentage of three-drug cured cases. Survival is defined as time before tumor grows to 10^13^ cells. This number is intended to represent the sum total of the cell numbers in a very large number of metastatic lesions, since most patients succumb to widespread metastatic disease. Cure is defined as total elimination of disease. Note that because the 3 drug simulation contains more states and allows doubly resistant cells at time zero, results from it cannot be directly compared to results from the two drug simulation

**Fig. 2:**
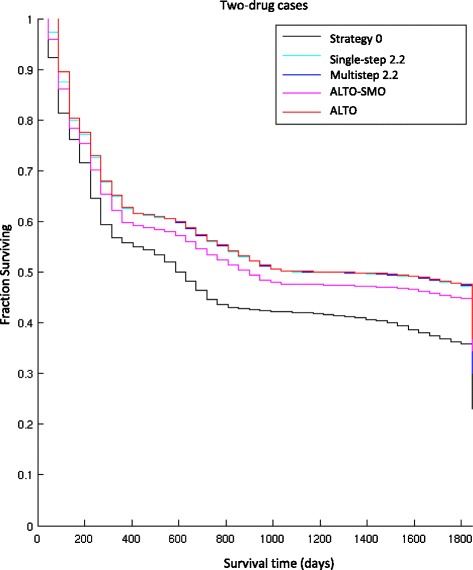
Kaplan-Meier survival curves of 5 treatment strategies from simulations for 2-drug cases. The 5 treatment strategies include: (1) strategy 0, the current personalized medicine strategy, (2) single-step strategy 2.2, (3) multi-step strategy 2.2, (4) ALTO-SMO and (5) ALTO. Other than strategy 0, the curves are largely overlapping

**Fig. 3:**
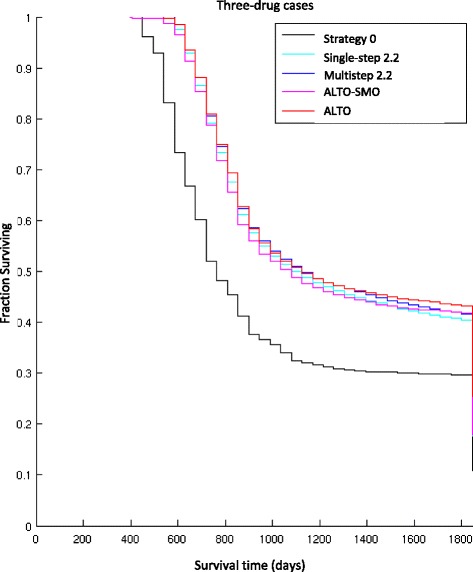
Kaplan-Meier survival curves of 5 treatment strategies from simulations. for 3-drug cases. The 5 treatment strategies include: (1) strategy 0, the current personalized medicine strategy, (2) single-step strategy 2.2, (3) multi-step strategy 2.2, (4) ALTO-SMO and (5) ALTO. Other than strategy 0, the curves are largely overlapping

The performance statistics in Table 1 include median survival times, percentage of virtual patients surviving for 5 years, and percentage of patients cured. Survival time is defined as the time the tumor burden is maintained at less than 10^13^ cells. In agreement with [2], we find dynamic precision strategies are significantly superior to the current precision medicine strategy 0 in all parameters. Multi-step heuristics and ALTO do not show a significant median survival benefit relative to single-step strategies across the virtual populations, and their survival curves look very similar (Figs. 2 and 3).

**Fig. 4:**
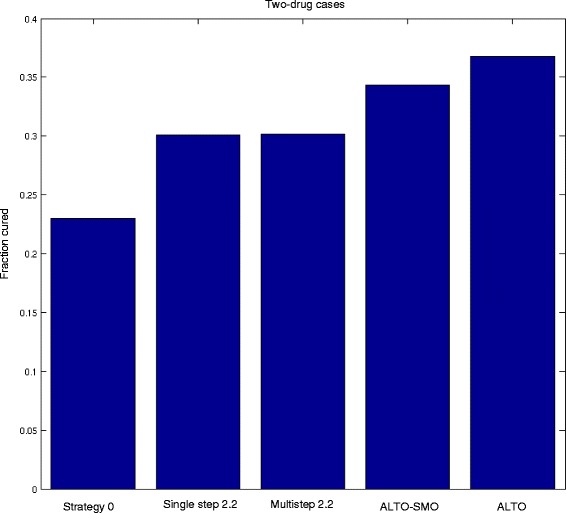
Cure rates for 5 treatment strategies from simulations for 2-drug cases. The 5 treatment strategies include: (1) strategy 0, the current personalized medicine strategy, (2) single-step strategy 2.2, (3) multistep strategy 2.2, (4) ALTO-SMO, and (5) ALTO. ALTO significantly enhances the cure rate

**Fig. 5:**
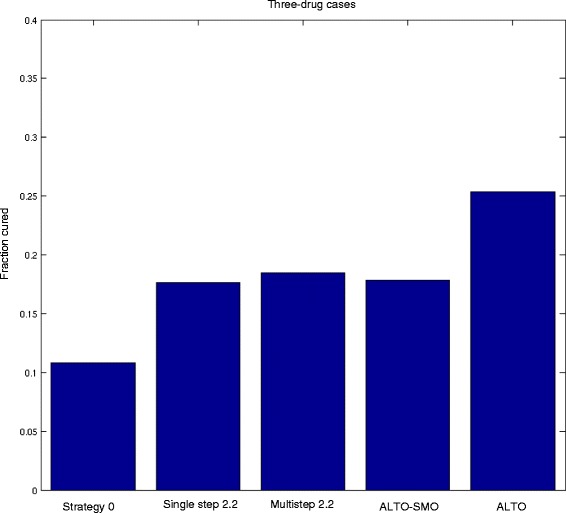
Cure rates for 5 treatment strategies from simulations for 3-drug cases. The 5 treatment strategies include: (1) strategy 0, the current personalized medicine strategy, (2) single-step strategy 2.2, (3) multistep strategy 2.2, (4) ALTO-SMO, and (5) ALTO. ALTO significantly enhances the cure rate

In contrast, when looking at cure rates, where cure is defined as elimination of all tumor cells. we find that planning with a time horizon up to 5 years offers significant benefits (Table 1, Figs. 4 and 5). In the two drug system, the single- and multi-step heuristics 2.2 offer approximately a 30 % cure rate in this curable population, whereas ALTO with a five year planning period increases the cure rate to 37 %. Similarly in the three drug system, the single- and multi-step heuristics provide an approximately 18 % cure rate, compared with 25 % using ALTO (Table 1, Figs. 4 and 5). The cure rate is substantially higher for all dynamic precision medicine approaches than for the current precision medicine strategy. The results are similar for other dynamic precision medicine strategies (Additional file 1: Table S2, Supplementary Results). An increased cure rate is a highly clinically significant outcome.

In light of the importance of combinations [42], we asked whether ALTO could be useful in a setting where only sequential monotherapy was allowed. We found (Table 1, Additional file 1: Table S2, Table 3, and Additional file 1: Table S4, Supplementary Results) that the ALTO-SMO strategy gave overall comparable performance to single- and multi-step heuristics that allowed combinations. In the two drug system, ALTO-SMO gave shorter median survival but higher cure rates.

We note that the mean survival for both ALTO-SMO and ALTO will be underestimated due to censoring of these additional cured patients when the simulation is truncated at 5 years. This suggests that long range planning may be at least equally important as combinations in the treatment of cancer. The best results occur when long range planning, combination therapy, and high dose monotherapy are all utilized.

It is instructive to look at not just average performance but performance in individual patients, especially given that precision medicine involves customization of therapy. In oncology clinical trials, a minimally clinically significant benefit is often defined as a 25 % relative survival improvement with a minimum 60-day absolute improvement. Using this criterion, Table 2 lists the counts of virtual patients where single-step heuristic 2.2 is significantly superior to its multi-step counterpart or vice versa. There are far more significantly superior cases of multi-step 2.2 than for single-step counterparts. This is equally true for the other dynamic precision medicine strategies (Additional file 1: Table S3). Thus, a subset of patients can receive significant additional benefit from multi-step heuristics compared to single-step heuristics, while there is little downside to the multistep heuristics. By looking several moves ahead, a multi-step heuristic can overcome the myopic limitation of a single-step heuristic, resulting in highly significant individual benefits in selected cases.

**Table 2:** Cases where multistep strategy 2.2 is clinically superior to single-step strategy 2.2 and vice versa

Number of drugs	Multistep > Single-step	Single-step > Multistep
2	6199 (99.8 %)	13 (0.2 %)
3	18909 (99.99 %)	2 (0.01 %)

A clinically superior outcome must provide at least a 25 % relative improvement and 2 month absolute improvement in survival relative to its comparator strategy. 1. Note that because the 3 drug simulation contains more states and allows doubly resistant cells at time zero, results from it cannot be directly compared to results from the two drug simulation.

A similar analysis can be performed comparing ALTO to all the other strategies including single-step and multi-step heuristics, and is shown in Table 3 featuring a comparison to strategies 0 and 2.2, and in Additional file 1: Table S4, Supplementary Results. Here we see that when viewed at the individual patient level, ALTO over a five year course is superior to both single-step and multi-step heuristics, and also to ALTO-SMO. We note that all are markedly superior to the current personalized medicine strategy, strategy 0.

**Table 3:** Cases where ALTO is clinically superior or inferior to each indicated strategy

Strategy	Inferior 2	Superior 2	Inferior 3	Superior 3
Strategy 0 (Current precision medicine)	5 (0.003 %)	176718 (99.997 %)	1808 (0.2 %)	898155 (99.8 %)
Single step strategy 2.2	5 (0.08 %)	6378 (99.92 %)	5631 (14.5 %)	33093 (85.5 %)
Multi-step strategy 2.2	5 (2.7 %)	179 (97.3 %)	6247 (33.1 %)	12608 (66.9 %)
ALTO-SMO	5 (0.02 %)	32621 (99.98 %)	6313 (10.4 %)	54374 (89.6 %)

Strategy 0 is the current personalized medicine strategy: treat with the best drug for the largest clone and continue to treat until tumor worsening or relapse, then rebiopsy and repeat. Strategy 2.2: select/adapt treatment every 45 days using evolutionary dynamic model to minimize the likelihood of forming a cell simultaneously resistant to all the therapies at a future reference timepoint, unless the estimated tumor burden is 10^11^ cells or more. Single-step strategy 2.2: future reference timepoint for selecting treatments is 45 days, corresponding to the interval between treatment adaptations. Multistep strategy 2.2: future reference timepoint for selecting treatments is 225 days, or 5 times the interval between treatment adaptations (“thinking 5 steps ahead”). ALTO: Adaptive long-term optimization. ALTO-SMO: Adaptive long term optimization-serial monotherapy only. Inferior 2: the number of two-drug cases where the ALTO strategy is clinically inferior to each selected strategy. Superior 2: the number of two-drug cases where the ALTO strategy is clinically superior to each selected strategy. Inferior 3: the number of three-drug cases where the ALTO strategy is clinically inferior to each selected strategy. Superior : the number of three-drug cases where the ALTO strategy is clinically superior to each selected strategy. A clinically superior outcome must provide at least a 25 % relative improvement and 2 month absolute improvement in survival relative to its comparator strategy. Note that because the 3 drug simulation contains more states and allows doubly resistant cells at time zero, results from it cannot be directly compared to results from the two drug simulation.

### Examples of cases where multi-step heuristics or ALTO achieved highly significant benefit

**Fig. 6:**
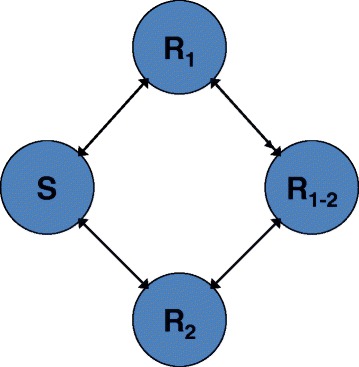
A minimal population dynamic model for a 2 drug system with four phenotypic states. S = sensitive cells. R_1_ = cells resistant to drug 1 and sensitive to drug 2. R_2_ = cells resistant to drug 2 and sensitive to drug 1. R_1-2_ = cells resistant to both drugs. Arrows indicate reversible genetic or epigenetic transitions between phenotypic states. Each phenotypic state may represent a cluster of related genotypes. Reproduced from [40] with permission

In this section, we present three examples of the value of long range planning: one case in which a multi-step heuristic outperforms the corresponding single-step heuristic, and two cases in which ALTO outperforms a multi-step heuristic, one in a two drug system and one in a three drug system. In order to clarify the examples, we will briefly review the evolutionary model for two non-cross resistant drugs (or drug combinations), illustrated in Fig. 6. More details of the model are given in the methods section. Four phenotypic states are illustrated in Fig. 6, corresponding to a 2 × 2 table of sensitivity and resistance to the 2 drugs. S cells are sensitive to both drugs 1 and drug 2. R_1_ cells are resistant to drug 1 and sensitive to drug 2. R_2_ cells are resistant to drug 2 and sensitive to drug 1. R_1-2_ cells are resistant to both available drugs/drug combinations and hence considered “incurable” with available drugs. The patient presents with a mixture of these subclones which evolves over time. We do not allow “incurable” cells at diagnosis since this state is not rescuable by any strategic manipulation of the available drugs. The arrows indicate somatically heritable transitions between the phenotypic states by genetic or stable epigenetic mechanisms, and the rates may differ for different transitions. All cells are growing exponentially, but their growth can be inhibited or reversed by the drugs in a dose dependent manner according to their drug sensitivities. At each 45 day timepoint, the physician utilizes the evolutionary model and a strategy, as described in the introduction, to choose an optimal therapy, which may consist of full dose drug 1, full dose drug 2, or a 50-50 reduced dose mix of the two. In the three drug case, we have 8 phenotypic states representing a 2 × 2 × 2 matrix of states of sensitivity and resistance to the 3 non-cross resistant drugs, with analogous nomenclature and analogous transitions between the states. Incurable triply resistant R_1-2-3_ cells are not allowed at diagnosis. At each 45 day timepoint, the physician may choose one of 7 options: full doses of drugs 1, 2, or 3; 50-50 reduced dose mixes of the 1-2, 1-3, or 2-3 combinations, or a 33-33-33 reduced dose mix of drugs 1, 2, and 3.

**Fig. 7:**
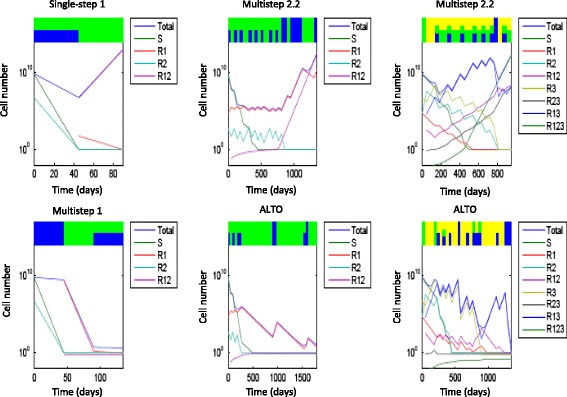
Treatment sequences for example cases with a significant outcome difference based on strategy horizon. Left: single-step vs. multi-step strategy 1 for 2 drugs. Middle: multi-step strategy 2.2 vs. global optimization for 2 drugs. Right: multi-step strategy 2.2 vs. global optimization for 3 drugs. In each example, the dosage sequences and population dynamics of two strategies are demonstrated in the top and bottom rows. Horizontal axes denote time, and vertical axes denote the population size in log scale. The dosage combination in each period is displayed by color bars on the top. The height of each color bar is proportional to the dosage of each drug. Blue: drug 1, green: drug 2, yellow: drug 3. The temporal response of each subpopulation size is displayed as a curve according to the legends by each figure

The three examples illustrating the potential value of long range planning are shown in the three columns of Fig. 7. Each patient presents with an initial total population of 10^9^ cells, representing a 1 cm^3^ lesion. Each example applies to a particular virtual patient with a particular population of subclones, drug sensitivities, and rates of genetic and epigenetic evolution.

A drastic outcome difference between single-step and multi-step strategies of the same heuristic is illustrated in an example in the left column of the Fig. 7. In this example, drug 2 is far more effective than drug 1 on sensitive subclones (sensitivities 0*.*8 and 150 for drugs 1 and 2 respectively). Yet a minority initial *R*_2_ population (5 × 10^6^) and a high transition rate to drug 1 resistance (4*.*5 × 10^−5^) cause the patient to be vulnerable to development of an incurable *R*_1-2_ cell from an R_2_ precursor if the *R*_2_ subpopulation is not prioritized for therapy. Single-step strategy 1 first administers the two-drug combination to minimize the total population. The sensitive and singly-resistant subclones are nearly eradicated, but the *R*_1-2_ population emerges from the initial *R*_2_ subclone. The patient dies in the second treatment period from *R*_1-2_ growth.

In contrast, multi-step strategy 1 first administers a full dosage of a less effective drug 1 to more rapidly control the *R*_2_ population. The total population at the end of period 1 far exceeds the corresponding total population for single-step strategy 1, which begins with a two-drug combination. However, this larger population is dominated by *R*_1_ and thus can be eliminated by drug 2 in the subsequent periods. Thus, application of multi-step strategy 1 results in cure despite an initial move that appeared to be inferior. The example illustrates the principle that singly resistant subclones can be very dangerous if they are able to rapidly evolve multiple resistance. Marked variations in resistance acquisition rates between subclones are to be expected due to differing degrees of genetic instability conferred by different genetic instability mutations [29] and differing numbers of ways to acquire the resistance phenotype [2]. Differing profiles of variability from different genetic instability mutations were predicted to exist within different cells of the same individual cancer [4] and this has recently been confirmed in lung cancer [43, 44].

In the second example, we compare ALTO to multistep heuristic 2.2 in a two drug system (middle column of Fig. 7). In this example, drug 2 is more effective than drug 1 on sensitive subclones (sensitivities 0*.*06 and 0*.*23 for drugs 1 and 2 respectively), and the transition rate of acquiring drug 1 resistance (4*.*6 × 10^−5^) is higher than the rate of acquiring drug 2 resistance (10^−7^). The initial tumor is heterogeneous, containing 1 × 10^9^ S cells, 5 × 10^4^ *R*_1_ cells, and 5 × 10^2^ *R*_2_ cells. Multistep strategy 2.2 minimizes the risk of *R*_1-2_ emergence when the total population *<* 10^11^. Initially *R*_2_ represents a higher risk than *R*_1_ due its much more rapid rate of acquiring resistance to drug 1 and becoming an incurable doubly resistant cell. Multistep strategy 2.2 thus allocates more dosages to curb the *R*_2_ population. By the end of the first episode (5 treatment periods or 225 days), strategy 2.2 yields a low *R*_2_ population (11) but a high *R*_1_ population (4*.*1 × 10^5^). The risk from the high *R*_1_ population is no longer negligible, hence strategy 2.2 administers several two-drug combinations in the following episode. Subsequently, the doctor is busy switching treatments to put down one subclone but elevate another. Eventually the *R*_2_ population reaches a critical value, *R*_1-2_ cells arise from *R*_2_ and the patient dies in 1350 days.

In contrast, ALTO adopts a different strategy. Initially it allocates more dosages to control the *R*_2_ population. By 270 days *R*_2_ is eliminated while *R*_1_ rises to 9 × 10^5^. It then administers a full drug 2 dosage for several periods to lower the *R*_1_ population. Eventually all subpopulations are eradicated and the patient is cured. A reduction in tumor diversity by fully eliminating a single sub-population may be critical in this example.

The final example (right hand column of Fig. 7) compares ALTO with multi-step strategy 2.2 in a three drug system. In this example, drug 1 has a stronger effect on sensitive cells (sensitivity 0.28) than drugs 2 and 3 (sensitivities 0.08). Initially there are 1 × 10^9^, 5 × 10^4^ *R*_1_ and *R*_2_ cells and 5 × 10^6^ *R*_3_ cells. The transition rates of acquiring resistance to drugs 1 to 3 are 10^−11^ *,* 10^−3^ and 10^−5^ respectively.

Multi-step strategy 2.2 minimizes the risk of emergence of incurable multiply resistant cells. *R*_1_ cells carry greater multiple resistance risk than *R*_2_ and *R*_3_ cells with the above parameters as the latter have a slow rate of *R*_*1*_ resistance acquisition. Multistep strategy 2.2 initially administers a full dosage of drug 2 in the first period, followed by full dosage of drug 3 in the next two periods, both of which reduce the R_1_ population. In the fourth period, strategy 2.2 administers a three-drug combination to reduce subpopulations with non-negligible sizes (*S, R*_*2*_ *, R*_*3*_ *, R*_*2-3*_). At the end of period 5, all the subpopulation sizes following the multistep strategy are smaller than they would have been following the single-step strategy (not shown). However, the reduced populations only slightly delay emergence of the multiply resistant subclones. The survival time extends from 900 days with single-step strategy 2.2 to 945 days with multistep strategy 2.2.

ALTO has the same dosage combinations in the first three periods as multistep strategy 2.2. In contrast to multi-step strategy 2.2, ALTO administers a third consecutive full dosage of drug 3 in period 4. This treatment substantially increases *R*_*3*_ and *R*_*2-3*_ populations, yet reduces the *R*_1-2_ subpopulation from 40 to 10. This apparently inferior move has important consequences, as *R*_1-2_ is successfully controlled in the long term by ALTO yet steadily increases under multistep strategy 2.2. As we saw earlier, R_1_ cells and their derivatives are more dangerous in this virtual patient due to the more rapid ability to acquire resistance to drugs 2 and 3. A highly complex series of maneuvers eventually results in cure. Visual inspection of the diagram suggests that the multistep strategy 2.2 recommendations have a certain regular periodicity which the tumor eventually overcomes, whereas ALTO has a more complex recommendation. Both strategies include highly complex adaptive patterns of interleaved monotherapy and combination periods.

## Discussion

Prior work [2] has shown, within a single-step optimization paradigm, that dynamic precision medicine strategies explicitly considering intratumoral heterogeneity and evolutionary dynamics can in principle confer dramatic improvements in mean and median survival as well as greatly enhanced cure rates for metastatic cancer patients.

Yet, even the dynamic precision medicine strategies of our prior work are myopic in that they are single-step heuristics with an explicit planning horizon of 45 days. In this paper, we have examined treatment strategy horizons of up to five years, in up to 40 individual 45-day maneuvers, unprecedented to our knowledge. The strategies have been examined for a wide variety of conditions comprehensively surveying all potentially curable initial conditions which appear to be consistent with the literature and clinical experience.

We have shown that longer term planning leads to additional improvements in outcome, which, while small on average, are of great importance for a subset of individual patients. In particular, there is a significant increase in cure rate, an outcome highly valued by patients. The popularity of high dose chemotherapy protocols with bone marrow transplantation demonstrates that patients will actually risk mortality from therapies in order to enhance their chance of a cure. However, applying long range planning to current therapies does not appear to be associated with any downside risk based on the results of this study. The average benefit of long-term planning is likely underestimated in the study in that survival of cured patients is truncated at 5 years (the length of the simulation). The relative frequency of different parameter combinations, representing different virtual patients, is unknown, and thus all parameter combinations were weighted equally. Thus, the magnitude of benefit observed in this study may differ from the benefit observed in a real population. The lack of downside risk does robustly indicate a net benefit, however. Further work is needed to identify the subset of patients who benefit from long range planning, increasing the average benefit further according to the principles of precision medicine.

The advantage of thinking ahead is also evident from the comparison of numbers of cases where one method is significantly better than another, in which a 5 year strategic planning horizon outperforms a 225 day planning horizon, which in turn outperforms the original 45 day horizon. Long term outcomes appear to be more likely with long term planning.

These findings have significant implications for the future of clinical research as well. Whereas computational experts have long known that “greedy algorithms” which seek short term gains are inferior optimization tools to those with longer time horizons [45], we find the increasing use of short term endpoints such as tumor shrinkage to adaptively govern randomization of patients in master protocols that match multiple therapies to multiple biomarker-defined patient subsets simultaneously [46, 47]. Master protocols are an important step forward in that they are a highly efficient way to match biomarker defined subset to therapies. Such matching is essential, as one cannot play chess without first learning the rules. However, short term endpoints may not always correlate with long term benefits, and we prefer master protocols which govern adaptations based on longer term outcomes or on short term endpoints that have been extensively validated as correlating with these endpoints. The work discussed herein provides biological reasons why adapting based on short term responses of the largest subclone, leading to tumor shrinkage, may be misleading in some instances.

In the current study, in which complete information is assumed, the long term strategies are executed as designed for their full planning horizon. In real applications with incomplete information, the long-term strategies would be updated every 45 days based on a comparison of predictions and results, allowing the system to “learn” based on progressive Bayesian updates to probability distributions of parameter values.

The superiority of ALTO, which of necessity must rely on a mathematical approximation, to heuristic approaches which incorporate clinical and biological intuition into the computational framework, raises complex issues. In this simulation, complete information about parameters, a completely accurate evolutionary model, and frequent access to tissues are assumed, and yet it is well known that these elements will not be available in real situations. In the face of these obstacles, it would be premature to assume that a purely theoretical approach could fully supplant biological and clinical intuition. Regardless of the computational approach used, its recommendations need to both inform and be informed by biological and clinical principles. The development and testing of heuristic algorithms against experiment allows this to occur, and therefore heuristic algorithms may have an important role. Further research is needed to develop methods which can plan further ahead and still be intuitive.

From a translational perspective, efforts should be made to collect patient materials from rapid autopsy to work backwards from long time horizons [2]. Moreover, translating these ideas in real clinical situations optimally will require improvements in other technologies as the approach ideally demands serial sampling of tumors followed by detection, isolation, and molecular and phenotypic analysis of rare sub-clones to determine their growth rates, drug sensitivities, and heritable phenotypic transition rates. Relevant technologies include immortalization of patient materials [48, 49], circulating tumor cells [50], plasma DNA analysis [51], specific imaging probes [52], single cell sequencing [53], and duplex DNA sequencing for identifying rare sub-clones [54]. However, each of these have limitations and therefore it is anticipated that some key model parameters will not be directly measurable in given individuals and need to be simulated as probability distributions based on population data [40]. We envision that the initial probability distributions of parameter values would be provided from population databases, and iteratively refined in a Bayesian fashion in individual patients based on subsequent observations in that individual. The resulting optimal strategies must incorporate a probabilistic analysis of possible outcomes.

The need to comprehensively evaluate multiple parameters as probability distributions limits the feasible complexity of the core model, as the computational complexity expands exponentially with the number of unmeasured model parameters. More complex and “realistic” models will result in increasing challenges to measure the relevant parameters in patients.

The simplicity of the core model analyzed herein is therefore essential. We also note that the current precision medicine paradigm,with its static matching approach, has produced meaningful patient benefits, and we believe that a first order approximation to precision medicine incorporating dynamics can be similarly beneficial without representing all the known and unknown features of cancer.

The core model does not explicitly account for numerous complexities of real cancers and cancer therapy, including inhomogeneous biodistribution of therapies into tumor tissue, the distinction between driver and passenger mutations, non-heritable adaptations, tumor cell dormancy, competitive and cooperative interactions between sub-clones, and interactions with the host stroma and immune system [55–59]. However, in actual application, the model should be linked with knowledge sources such as measurements on ex vivo tumor tissues, cell line banks, population molecular and clinical data, theoretical pathway and network knowledge, and functional genetic screens [11, 60–62]. These sources should be linked to the core model in a modular fashion to inform the probability distribution of parameters based on laboratory or population data, and this is an important challenge for future research [2, 40].

For example, the drug sensitivity phenotype of a heritable state will not have a single fixed value, but rather a probability distribution based on such factors as inhomogeneous biodistribution of the therapy into tumor tissue, spatial heterogeneity of the tumor microenvironment, tumor dormancy, and non-heritable adaptations. We hypothesize that non-heritable adaptations will operate on a faster time scale than genetic evolution, and the former will be responsible for primary resistance and early relapse, whereas the latter will govern late relapses. If this hypothesis is correct, multiscale integrated models of non-heritable adaptations and genetic evolution could be more readily developed in selected instances by separating them on the basis of timescale. We further hypothesize that the available non-genetic adaptations and their fitness costs will ultimately depend on the genetic endowment of a cell, such that a genetic alteration may lower the fitness cost of initial non-genetic resistance in some cases. Further, each phenotypic state actually represents many underlying heritable states as well, and the transition rates between phenotypic states will be the sum of rate constants from many individual transition mechanisms [2], for example the multiple mechanisms of resistance development to epidermal growth factor receptor tyrosine kinase inhibitors in lung cancer [63]. Finally, cooperative and competitive interactions between subclones are readily added to the core model itself by replacing the scalar net growth rate with a net growth rate matrix with cross terms. In all of these cases, the additional complexity would be added to the core model and/or linked models only if supported by experimental data as well as clinical data in relevant populations, and the ability to measure the resulting parameters in the majority of individual patients would be preferred.

Our model defines “states” in terms of drug efficacy, and each such state includes multiple molecular configurations. There are mathematical models that capture refined states of molecular alterations, for example multistage mechanisms such as increase or decrease of copy numbers of cancer-related genes [64–66]. Such models can provide a more precise description of specific mechanisms, yet substantial expansion of model complexity also makes parameter estimation, treatment optimization, and large-scale simulations much less tractable. If there is a monotonic relation between copy numbers and drug resistance, and state transitions are relatively homogeneous (e.g., the transition probabilities from 2 to 3 copies and from 3 to 4 copies are in the same scale), then our model is a reasonable approximation.

The approach herein also relies on a continuous approximation rather than a stochastic simulation approach. Given the large number of treatment sequences to be evaluated and the desire to add additional complexities, the computational cost of stochastic simulation may be prohibitive. While a continuous approximation may not delineate the variability in outcomes under identical conditions, it has been shown to accurately predict the average outcomes in the case of genetic evolution of drug resistance [67].

Several authors have argued for the importance of combination therapy in addressing the complicated and dynamic nature of cancer [42, 68–71]. Moreover, combination therapy has been highly successful against the human immunodeficiency virus [72], which has a uniformly rapid rate of evolution but a much less complex genome than a eukaryotic cell.

We agree with several key conclusions from these authors. Combinations are an essential component of successful cancer therapy in our view. When it is possible to give the desired combinations in full dose, this is likely to be superior. Sequential monotherapy *by the current personalized medicine strategy* is clearly problematic. A sufficient number of non-cross-resistant agents or combinations are required to deal effectively with the diversity and dynamic nature of cancer. However, our work also differs in several important respects, and ultimately leads to a much more complex recommendation involving rapidly interleaved pulses of full dose monotherapy and combinations specifically tailored to individual population structure and dynamics.

First, we consider the frequent need for dose reduction in combination due to toxicity. Occasionally dose reduction is not necessary but often it is, and in general if we want to select the combinations for optimal therapeutic effect we will not always be able to simultaneously select for non-additive toxicities. The need for dose reduction in combination therapy creates strategic dilemmas. The genetic complexity of cancer, far exceeding that of the human immunodeficiency virus (HIV) due to the larger number of genes, exacerbates these dilemmas. We note that each genetically or epigenetically distinct sub-clone likely requires a combination for its eradication due to non-heritable resistance mechanisms such as feedback loops. A cancer with multiple heritably distinct sub-clones would likely require combinations of combinations, and these higher order combinations would likely not be feasible to administer simultaneously at meaningful dosages. (In our formulation, “monotherapy” may mean a lower order synergistic combination directed against a single subclone). In addition to reduced pharmacodynamic effect, lower dosages may impair biodistribution into the tumor space [73].

Second, in contrast to previous authors, we allow *each subclone* to have different baseline rates of genetic change, and vary the overall transition rates for each subclone independently over 8 orders of magnitude, taking into account the possibilities of multiple and differing genetic instability mutations in different sub-clones and of heritable change by epigenetic mechanisms [4, 29, 44], in addition to the varying number of loci associated with different phenotypic changes which are common to our model and that of Bozic et al. [42]. Thirdly, we consider a much larger number of initial conditions across the parameters in general, comprehensively exploring parameter space relevant to oncology. Finally, instead of simply comparing long term monotherapy to combinations according to the current personalized medicine paradigm, we consider a very large number of complex treatment sequences. (“monotherapy” meaning single or combination treatment against a single heritably distinct subclone).

Accordingly, our recommendation for the role of combinations depends on the initial conditions and dynamics of each individual patient as well as the ability to deliver the relevant therapies in combination at full dosage, and their dose-response curves, synergy, and antagonism. Elaborate interleaved sequences of combinations and monotherapy are shown to be optimal in some cases. In other cases, part of the optimal treatment sequence involved rapid reduction in tumor diversity via sequential focused elimination of sub-clones using pulses of high dose “monotherapy”. Sequential reduction of tumor diversity narrowed the cancer’s options, backing it into a corner. Increased diversity has been shown to be associated with increased risk of tumor progression [74]. Diversity of therapy is maximized with complex patterns involving a large number of therapies, even when simultaneous administration of higher order combinations is not possible, by rapid interleaving sequences reassessed every 45 days.

Other authors have pointed out that high intensity therapy with the intent of complete tumor eradication may maximize selection pressure for resistance development. [75, 76] and recommend less intensive therapy. We find this concern particularly appropriate in the case of the current personalized medicine strategy that maintains a constant therapy as long as the patient is benefiting, where benefit is defined as lack of clear tumor worsening. This produces a smooth, predictable fitness landscape on which evolution to resistance is straightforward. In contrast, the complex and varied sequences of therapies discussed both in [75] and herein create unpredictable fitness landscapes. Evolution on jagged, unpredictable fitness landscapes is far more difficult [77].

In the current work, we have also shown equivalent results in a system of three non-cross-resistant agents, and the lessons for therapeutic strategies appear to be similar to the two drug case in that similar dynamic precision medicine strategies were optimal in both cases. However, absolute survival times of the two and three drug simulations cannot be directly compared since in the three drug simulation, in contrast to the two drug simulation, doubly resistance cells were permitted at time zero. Furthermore, in the three drug case the cancer was given additional genetic complexity (8 states rather than 4) to allow it to escape three drugs. This illustrates the point that the number of drugs required to confer clinical benefit or cure depends on the underlying genetic complexity of the cancer. This complexity is likely to be very high given current theoretical and experimental knowledge.

The best multi-step heuristic in both two and three drug systems, strategy 2.2, attempts principally to prevent the development of multiple resistance unless the tumor burden is large enough to be immediately threatening. The importance of preventing multiple resistance recalls the earlier work of Goldie and Coldman [68], and confirms both our earlier work [2] and the more recent study by Bozic et al [42].

## Conclusions

Therapy planning with a long strategy horizon provides significant benefits and previously unrealized cures to selected patients. Optimal strategies incorporate both combinations and high dose “monotherapy”. Similar principles apply in both two and three drug cases. These results have significant implications for future precision medicine paradigms as well as clinical and translational research methods.

## Methods

The work herein utilized a population dynamic model of tumor growth (Fig. 6 and Additional file 1: Supplementary Methods) and a formalization of the current personalized medicine strategy and 5 dynamic precision medicine strategies as single-step heuristics (Additional file 1: Table S1) [2]. The strategies, which were updated every 45 days, used an evolutionary model to predict the future state at the end of the 45-day interval, choosing the treatment which was predicted to either minimize the total cell number or the likelihood of forming a doubly resistant cell. The strategies differed in how they used the data to prioritize among these two goals.

The evolutionary model (Fig. 6) was a focused minimal model with two **non-cross resistant** targeted “drugs” (may be combinations) each optimal for a particular subset of heritable somatic variant states. Each heritable state corresponded to a different resistance profile due either to explicit resistance mutations among the “passenger mutations” or to a partially overlapping set of oncogenic mutations, leading to differing pathway addictions [7, 13]. There were 4 phenotypic states representing *2* × *2* possibilities of sensitivity and resistance to the two agents/combinations in the two drug simulations and 8 phenotypic states representing *2* × *2* × *2* possibilities in the three drug case. The model featured exponential growth and first order heritable transitions between the states, as well as dose dependent reduction in net growth rate by drugs. Virtual patients had an evolving mixture of cells rather than a single consensus clone.

The model assumed that non-cross resistant therapies could be identified to address the different heritable states with the exception of one “incurable” multiply resistant state which was assumed to not be pre-existing. Each therapy may itself be a single agent or combination but is directed at a single heritable somatic state. Importantly, if multiple drugs were given in combination, the dose was reduced due to toxicity, which is often the case in actual practice. If full doses of all agents can be given simultaneously, simultaneous combinations are optimal [42], but when that is not the case a more complex strategic dilemma occurs.

Resistance to the two (or three) non-cross resistant therapies is assumed to be acquired stepwise. If resistance to all therapies utilized can occur in a single step, therapy is unlikely to be effective regardless of strategy [42], and therefore these scenarios are of less interest for the present work.

The central equation expressing these points is given below. The instantaneous accrual rate of each subpopulation is the intrinsic net growth rate plus the heritable transition rates to it from other subpopulations, minus the drug-induced cell death rate. Given *K* cell types and *D* non-cross resistant drugs (each of which may in fact be a combination directed at a single heritable somatic state), their population dynamics can be concisely expressed as a vector differential equation:

1 $$ \frac{dX}{dt}\kern0.5em =\kern0.5em \left[\left(I+T\right){\mathit{\mathsf{g}}}_0\kern0.5em -\kern0.5em dia\mathit{\mathsf{g}}\kern0.5em \left({S}_a\kern0.5em d\right)\right]U\left(X(t)\kern0.5em -\kern0.5em 1\right)X(t) $$

where a *K × 1* vector *X(t)* denotes the size of each subpopulation, *g0* denotes their intrinsic growth rate (the model may be easily generalized to allow different growth rates for different phenotypic states), *I* is a *K × K* identity matrix, and *T* a *K × K* heritable transition rate matrix. A *D × 1* vector *d(t)* denotes the normalized dosage of each non-cross resistant drug where the sum over all drugs equals to one. Requiring the normalized sum of the dosages to equal 1 expresses the need for dose reduction in combination, but in a real application the allowed dosage combinations would be taken from Phase 1 clinical studies. A *K × D* matrix *Sa* denotes the sensitivity of each drug on each cell type. The current study is based on the drugs enhancing the death rate of cell populations, but the model may be easily generalized to include drugs which slow the growth rate instead. *U(X(t) − 1)* is a component-wise step function. It sets the growth rate to zero when the subpopulation size is below a single cell, preventing exponential growth from a negligible subpopulation.

Additional details are provided in Additional file 1: Supplementary Methods.

### Experimental basis of parameter selection

Each virtual patient represented a unique parameter set of net growth rates, drug sensitivities, initial sub-populations, and genetic/epigenetic transition rates between the heritable states. A large number of parameter configurations (approximately 760,000 for the two drugs cases and 1.7 million for the three drug cases) were considered based on a comprehensive review of the clinical and experimental literature, and the virtual patients represent a thorough sampling of possible oncology scenarios, limiting to “curable” cases where both drugs are capable of producing net negative growth rates for their respective populations when given at full dose. The comprehensive sensitivity analysis over a very large number of virtual patients differentiates this work and the current study from other efforts in this field. A variety of sources were used to ensure that the parameter ranges were realistic as well as sufficiently broad to encompass all likely oncology scenarios. These included preclinical and clinical literature as well as experience of one of us in oncology patient care and clinical research, comprising several dozen experimental oncology therapeutics in most major tumor types and thousands of patients over several decades.

For example, the most rapid tumor growth rates were informed both by the preclinical studies of fully cycling cells [30, 31] and clinical observations of a Burkitt’s lymphoma patient. The slowest tumor growth rates were derived from observations of 8000 men in a clinical research study of bicalutamide adjuvant therapy of prostate cancer [32] led by one of us, and are also in accord with growth rates observed in a study of localized pancreatic cancer [33].

The phenotypic transition rates were varied over 8 orders of magnitude from 10^-11^ to 10^-3^. The lowest rate assumes the low rate of genetic change measured preclinically in stem cells [34], and that only one single base in the genome governs the phenotype and must be mutated for a transition to be observed. It is in accord with observed mutation burdens in retinoblastoma [35]. The highest transition rates incorporate maximal increases in genetic instability for point mutations that have been observed preclinically [29, 36], the likelihood that a single amino acid change will alter protein function [37], and the possibility of multiple sites in the genome, alteration of which can lead to the phenotype. It is also sufficient to account for a scenario in which 10 independent resistance mechanisms exist and the cells additionally have a severe chromosomal instability defect [38]. This broad range of phenotypic transition rates is also compatible with the broad range of transition rates that fit a locally advanced pancreatic cancer dataset [33].

We note that the results reported in Beckman, Schemmann, and Yeang [2] concerning benefit of dynamic precision medicine strategies have been shown to apply throughout this very broad parameter space. That is, patients who benefitted from dynamic precision therapy do not cluster in a localized region of this space.

Additional detail on parameter selection is provided in the Additional file 1 to [2].

### Multi-step extensions of heuristics

The strategies in Table 1 are *single-step heuristics* that propose dosages for the next treatment period only. They are myopic in that treatment sequences that are beneficial in the long run but suffer from short-term losses will be excluded. In this work, strategies 1-3 were extended to design treatment sequences of multiple periods. Designing a treatment sequence with a fixed number *n* of look-ahead periods can be viewed as traversing a decision tree illustrated in Fig. 1. Each node denotes the population structure at the beginning of a treatment period, and the 2^*D*^ − 1 links emanating from this node denote the possible dosage combinations administered during the subsequent period (where *D* is the number of non-cross resistant drugs or drug combinations). The root encapsulates the initial population structure, and terminal (leaf) nodes denote the states where either their depths reach the look- ahead period *n*, the patient is cured (each subpopulation size *<* 1) or the patient dies (total population size exceeds the mortal threshold 10^13^). All possible *n*-step treatment sequences are represented as paths of length *n* in the decision tree.

Multistep extension of heuristic treatment strategies is realized by a branch-and-bound algorithm on decision trees [78]. At the beginning of each of the *n* treatment periods, a decision tree of subsequent possible *n*-step treatment sequences is generated. The algorithm traverses all paths along the decision tree and selects the one whose terminal population structure either vanishes (each subpopulation has *<* 1 cell) or satisfies the criteria stipulated by the heuristic strategy. To reduce unnecessary search a subpath is discarded when the population structure of an intermediate node exceeds the bounds of both total cell number and multiply resistant cell number established from previously traversed sequences at the same node depth. A detailed description of the algorithm is reported in Additional file 1: Supplementary Methods.

### Adaptive long-term optimization (ALTO) over treatment sequences

Long range optimization of treatment sequences is challenging. One has to construct the treatment decision tree with a depth equal to patients’ life spans or the maximum monitoring time, and find either the longest path or the path leading to cure. With 45-day treatment periods and a 5 year strategy horizon, employing fixed dosage combinations, there are 3^40^ = 1*.*2 × 10^19^ paths for two-drug cases and 7^40^ = 6*.*4 × 10^33^ paths for three-drug cases. Exhaustive evaluation is clearly intractable.

Two approximations are utilized. The first is a branch-and-bound method for pruning the tree. The survival corresponding to the population structure in each node of the tree is bounded from the top and the bottom. The upper bound of survival is given by the population dynamic model with full dosages of all drugs simultaneously. The lower bound of survival is that associated with the best available allowed static treatment sequence (taking toxicity into account) for the whole 5 year period without adaptation, as determined by the population dynamic model. A sub-path in the decision tree is inferior to a previously traversed sub-path if its upper survival bound is less than the lower survival bound of the previously traversed sequence. Inferior sequences are discarded.

The second approximation is building the tree from sub-trees of shorter length. The paths within a sub-tree are ranked by the geometric mean of their upper and lower survival bounds, and a limited number of top-ranking sub-paths are retained prior to building out the next incremental portion of the tree.

Both of these approximations are described and justified in detail in Additional file 1: Supplementary Methods.

### Simulation setup

The population dynamic model in Fig. 6 consists of 9 and 17 free parameters for two-drug and three-drug cases respectively, including the intrinsic growth rate, initial subpopulations, drug sensitivity ratios and heritable transition rates. Each parameter was varied over 7 possible values. The ranges of values were chosen to encompass the entire range of likely values over solid and liquid tumors based on experimental and clinical data [2]. To reduce the number of parameter configurations we applied filtering criteria to rule out the cases where the patient is cured or dies regardless of treatment strategies employed, and required, for this study of long range planning, that the patient be “potentially curable”, ie both drugs have the ability at full dose to cause progressive reduction of sensitive sub-opulations rather than merely slowing growth. 764104 and 1723116 parameter configurations passed these filters for two-drug and three-drug cases. For each parameter configuration, we implemented 11 treatment strategies, simulated their population dynamics, and calculated survival times under those regimens, where death is defined to occur when the total cell population exceeds 10^13^ from an initial population of 10^9^. Treatment strategies include the aforementioned five single-step strategies (Additional file 1: Table S1), four multistep extensions (strategy 0 excluded), and ALTO algorithms for all valid dosage combinations and for mono therapies alone (ALTO-SMO). The survival time of a patient cured before 1800 days was reported as 1845 days. Parallel processing utilized 23 Hewlett Packard (HP) DL360 G7 servers containing dual Intel(R) Xeon(R) central processing units (CPUs) E5520 with 2.27 gigaherz (GHz) and 24 gigabytes (GB) main memory. The total running time was 20 h for two-drug cases and 10 days for three-drug cases. Detailed descriptions of the simulation setup are reported in Additional file 1: Supplementary Methods.

## Reviewers’ comments

### Reviewer Report 1: Wendy Cornell, Principal Research Staff Member, IBM Watson Research Labs

Reviewer comments:

Reviewer summary –

The authors describe computer simulation studies of novel personalized and precise treatment strategies for cancer patients which build on and extend the initial strategy presented in their 2012 PNAS paper. Their strategy is provocative and challenges the current best practices which focus on the dominant clone and take a reactive approach to switching therapies. In contrast, the 2012 PNAS paper described a strategy which considers the best treatment at each step (45 day treatment period) to address the major threat, be it drug-resistant clone or overall tumor burden. The strategy anticipates and treats drug resistant clones, even when they do not represent the major clone in the tumor and are below detection level, before they can acquire resistance to a second drug. The model incorporates features such as intratumoral heterogeneity and evolutionary dynamics to represent the size and composition of the evolving tumor.

In this current manuscript the authors have added appropriate complexity to the strategy by thinking multiple steps ahead, much like a chess player, with both 5-step and 40-step (long term) approaches considered. This approach then leverages the vast amount of molecular and clinical knowledge which has been acquired over decades of cancer research. The simulations are carried out on a very large set of virtual patients whose characteristics fall into ranges drawn from literature data and the clinical experience of one of the authors. Results are compared for different versions of the strategy and improvements seen in cure rates. This new approach is well aligned with the call for improved personalized medicine and with recent advances in deep sequencing. The strategy is far from ready for clinical adoption, but the models and results motivate and provide a solid foundation for future clinical and translational research to identify best application of the existing mechanism-based cancer drugs. In summary, the work is highly original and significant and the virtual patients are modeled with valid characteristics. The details of valid clinical application should be explored and demonstrated in follow-up studies.”

Author response: *We thank Dr. Cornell for these comments*.

Reviewer recommendations to authors –

Major

The authors leverage literature data as well as their own significant clinical experience to select relevant parameter ranges for the simulations and note that it differentiates their work from others in the field. At the point this claim is made in the text a few references should be added.

Author response: *We have moved appropriate references from the methods regarding such parameters as transition rates. Some of the clinical information is from clinical experience treating patients and from leading clinical studies, however, and is not publicly available*.

Reviewer comment: Are the simulation inputs or results weighted according to relative frequencies of different parameter combinations? If not, then although the results are meaningful, it should be clarified that they do not suggest population outcomes for this set of virtual patients.

Author response: *we agree with this comment and have qualified the conclusions appropriately. The relative frequency of different parameter combinations is unknown. Importantly, since few patients appear to be harmed by the proposed approach, the conclusion that the approach represents a net improvement in cure rate is probably robust. However, exact quantification of the magnitude of the effect is confounded by the issue raised in this comment*.

Reviewer comment: How would the design of future clinical trials be impacted by the adoption of such alternative treatment strategies since there is no single strategy to test but rather many different individualized strategies?

Author response: *We see the approval of individual agents still requiring traditional trials, but these agents could then be tested post-approval in settings where their intended target is not the majority subclone. We also see the possibility of randomized trials where the same agents are utilized according to conventional precision medicine vs dynamic precision medicine, a higher order comparison independent of any individual therapy sequence*.

Reviewer comment: A fundamental concept promoted by the authors is that the best drug at a given stage is often not the best one for fighting the main clone population in the tumor, but rather one which is not quite as effective against the major clone but which is very effective against a minor clone population which is drug resistant. Since the population of the drug resistant clone is often below the detection level, how is it determined which drug is relevant to treat a pre-existing or later evolving resistant clone?

Author response: *Complete translation of these concepts certainly relies on optimal technologies for subclone detection and repeat access, which are emerging but are not yet optimized for solid tumors. However, we also propose formulating strategies based on incomplete data by probability weighted optimization. Based on population data and increasing molecular knowledge, we believe it will eventually be possible to define probability distributions for the existence of these states in populations that can be used for probability based optimization when information is missing. Repeated individual data over time will allow progressive refinement of probability based models in individual patients using Bayesian techniques*.

Reviewer comment: Minor

The 3-drug results are interesting and relevant to real world scenarios, but they are confusing when presented side-by-side in Tables 2 and 3 with the 2-drug results. The caveat that the two sets of results cannot be compared currently appears near the end of the Results section. Moving that caveat to appear sooner in the main text is recommended. The 3-drug results could even be moved to Additional file 1 to simplify the analysis since it is already very complicated. If the 3-drug results are left in the main text and in Tables 2 and 3, then the addition of a footnote to each table explaining why comparison is inappropriate is recommended.

Author response: *We have made the caveat concerning not being able to directly compare 2 and 3 drug simulations more prominent in the text and footnotes of Tables* *1* *,* *2* *and* *3* *. For the former Figs.* *2* *and* *3* *, we have made separate figures for 2-drug and 3-drug cases (now Figs.* *2* *,* *3* *,* *4* *and* *5* *). Extension to three drugs is important in that this level of complexity and likely higher will be required to deal with the diversity of tumors*.

Reviewer comment: Also, the addition of percentage values to Tables 2 and 3 in parentheses following the raw numbers is recommended.

Author response: *We have made the change requested*.

Reviewer comment: The rationale for selecting a 45 day time period should be explained.

Author response: *We have added an explanation. In a clinical trial, cancer patients often get a total estimate of tumor burden by computed tomography every 6 weeks. Thus, if a new therapy is started, its success is first judged after six weeks. In addition cancer treatments are often given in three week cycles which allow for recovery of the bone marrow and intestines from common toxicities, so that the patients are returning to be seen at three week intervals. Therefore, we simulated a situation with a new therapeutic decision every six weeks to coincide with the current schedule of other clinical activities. We rounded 6 weeks = 42 days to 45 days. The influence of the length of this period on our results is an interesting topic for future research*.

Reviewer comment: Many permutations are considered in this study and the nomenclature can be hard to follow. This reviewer had particular trouble remembering the differences between “global optimization” and “global monotherapy.” She suggests adapting the nomenclature to make the consistencies and distinctions between the different model features (single vs 2 or 3 drugs, single vs multi step planning, 5 vs 40(complete) steps) more explicit and clear.

Author response: *Per the suggestions from Dr. Cornell and other reviewers below, we have changed the terms “global optimization” to “adaptive long-term optimization (ALTO)” and “global monotherapy” to “adaptive long term optimization: serial monotherapy only (ALTO-SMO)”. We hope these terms are more intuitive and that the acronyms are helpful in aiding recall.*

Reviewer comment:

Addition of a figure showing the data and selected treatment for each time period for one or more examples would be very helpful.

Author response: *We engaged in further discussion with Dr. Cornell for clarification of her request. She agreed that Fig.* *5* *(now Fig.* *7* *) already serves this purpose*.

### Reviewer report 2: Marek Kimmel, Rice University

Reviewer summary –

I find this paper very interesting and important. Without an attempt to re-state the abstract, the authors show that while multi-step and global optimization of cancer strategies provide no significant average survival benefit, cure rates are significantly increased by global optimization. I think that the paper is very well-written and it is essentially suitable for publication in Biology Direct.

Author response: *we thank Dr. Kimmel for recognizing the importance of the paper*.

Reviewer recommendations to authors –

I find this paper very interesting and important. Without an attempt to re-state the abstract, the authors show that while multi-step and global optimization of cancer strategies provide no significant average survival benefit, cure rates are significantly increased by global optimization. I think that the paper is very well-written and it is essentially suitable for publication in Biology Direct. I list several items, which may be considered in a revision.

Line 121. “A situation was simulated in which two non-cross resistant drugs are available for treatment.” I would like to learn the authors’ opinion concerning how it can be determined that two anti-cancer drugs are non-cross resistant. I think this point is important, since it is conceivable that some types of resistance will not be limited to a single agent.

Author response: *Operationally, it means that no single molecular alteration conferring resistance to both agents simultaneously is known. Mechanisms of multiple resistance are known to exist for many classes of drugs, including those that work by different mechanisms. Like many features of this approach, increasing oncology knowledge will benefit this aspect.* In vitro*, one can treat with very high dose combinations and look for emergence of resistance in a forward mutation assay. It will not be easy to find truly non-cross resistant drugs. We have added some language to the introduction concerning these points*.

Reviewer comment: Line 150. “In the simulation, each patient started with a burden of 10^9^ cells (a single 1 cm3 lesion), and only if the total cell number increased to 10^11^ or more cells would strategy 2.2 choose the treatment that focused on minimizing the total cell number.” What will change in the conclusions of the paper if these numbers were varied, due to geometry of tumor, or other considerations?

Author response: *In Beckman, Schemmann and Yeang* [2]*, a related strategy with a threshold of 10* ^*10*^ *was evaluated the conclusions were similar. In the few cases where one threshold was better than another, the higher threshold was generally better. In real applications, total tumor reduction may be required for a variety of reasons, including local geometry as Dr. Kimmel points out. The best judge of this, as we have stated in this and other publications, will be the physician. The key to the strategy is that total tumor reduction is prioritized only when it is absolutely required. This is a very good point and suggestion for our future research*.

Reviewer comment: Line 289. “Survival time is defined as the time the tumor burden is maintained at less than 10^13^ cells.” This may be realistic in the cancers in which it is the primary tumor that kills the patient. However in many cancers, these are metastases that lead to terminal disease. Could you clarify this point?

Author response: *We regret that this critical point was not clear in the article. Actually we were not thinking of a single tumor killing the patient. Our approach is specifically designed for patients suffering from metastatic disease. By “tumor burden”, we mean total tumor burden across many lesions, uncountable widely spread metastases. A single lesion would have difficulty growing to 10* ^*13*^ *cells and still maintain a blood supply. Our experience agrees with Dr. Kimmel. Most patients will succumb to metastases, not a single large lesion. We have clarified that the approach is for patients suffering from metastatic disease, and that the number of tumor cells represents the total over all lesions*.

Reviewer comment: Line 309. “In the two drug system, global monotherapy gave shorter median survival but higher cure rates.” This does not appear clear to me. What is the relationship between these two measures of outcome?”

Author response: *The median reflects the 50 % point and may not correlate with cure rates when cures are less than 50 %. In global monotherapy, the lower median compared to global optimization (ALTO) must reflect inferior performance of global monotherapy (ALTO-SMO) compared to global optimization for some patients within the lower half of outcomes. Cure rates reflect the upper portion of the distribution of outcomes. Global monotherapy (ALTO-SMO), on the whole, performs surprisingly well, indicating that long range planning is as important as the simultaneous delivery of combinations. However, the most optimal approach is global optimization (ALTO) which incorporates long range planning and strategic use of both high dose monotherapy and simultaneous combinations as needed*.

### Reviewer report 3: Andrzej Swierniak, Silesian University of Technology

Reviewer summary –

“The manuscript is devoted to hot problems related to personalized anticancer therapy. The authors present outcome of the so called dynamic precision treatment strategies which take into account cancer evolution and intratumor heterogeneity. Using simulations on the populations of virtual patients the outcomes are compared for dynamic strategies in which optimization is performed in single 45 day step, 5 steps ahead and 40 steps ahead and two or three drugs are used. I believe that the topic and the results are of interest and worth publishing. The paper is clearly written and I accept the quality of language as well as in the presentation style. However I see a number of major and minor problems which should be overcome before the manuscript is accepted for publication.”

Reviewer recommendations to authors –

The paper is interesting, and contains original and clearly presented results. Nevertheless there are some problems that should be addressed before acceptance of the manuscript for publication.

Author response: *We thank Dr. Swierniak for recognizing the originality of the findings, as well as for his insightful and detailed analysis which provides important ideas for further research. We wish to make some general comments concerning Dr. Swierniak’s advice. The first point is that the primary purpose of this paper is to evaluate long term planning as an added feature in cancer therapy directed by evolutionary dynamics. As we state in our earlier paper* [2] *and the current one, the general principles we illustrate here should be applicable to any model based on evolutionary dynamics. While finding a complete evolutionary dynamic model of cancer is of great interest in general and for our own future research, it is not the focus of this paper. Secondly, as we have stated extensively in the discussion, the model we did employ does not (and is not intended to) fully represent all known complexities of cancer biology. In designing the model, we were guided by the principle of parsimony. Parsimony is essential for three reasons: 1. For the purpose of this study, the predictions of the model must be computable for a very large number of treatment sequences and over a vast combinatorial space of all adjustable parameters. Adding additional features to the model rapidly causes a combinatorial explosion of adjustable parameters. 2. A major goal of our research is actual translation to patient therapy, and we are actively engaged in discussions with experimentalists and clinicians in an attempt to do so. We agree with Dr. Cornell who stated above that even the current model is far from translation. In our view, the biggest obstacle is obtaining time series data from patients and measuring the parameters of our model in real time to inform patient therapies. Again, adding additional model features greatly exacerbates this problem. We have therefore designed what we believe to be a first order approximation to evolutionary dynamics. We believe this has the potential to help patients, in that even the zero order static matching of current precision medicine has had a clear positive impact for many patients. 3. The large number of theoretical models for cancer in part mirrors the large number of experimental models, each of them contrived to illustrate a particular aspect of the problem. In our experience in clinical development of experimental anticancer agents, the experimental data has rarely been predictive of clinical outcomes. We believe this is due to the fact that such models are specifically contrived, and each one represents only a small fraction of what is seen in a clinical population. That is, each patient will be different and require different customization of the simple generic model. In actual application, we would be interested in adding complexities to the model* ***if and only if they can be evaluated for their relevance and quantitatively characterized in individual patients in real time*** *, something which we hope will improve over time. In addition to this general concern, we believe current experimental models suffer from very low carrying capacity relative to the clinical situation, causing exaggerated perceived importance of competitive dynamics between subclones, as well as from small numbers of cells and limited observation time relative to clinical situations, resulting in underestimation of importance of rare genetic events*.

Reviewer comment: Major problems: 1) The authors seem to understand that drug resistance and other processes altering the behavior of cancer cells are driven by dynamical mechanisms but they describe them by classical mutation models (Fig. 4 in the manuscript). Multistage mechanisms including a gradual increase in number of discrete units (see e.g. (Harnevo and Agur, [65]), (Kimmel and Axelrod, [64])) which better describe such transformations lead to other class of compartmental models and dynamical properties of systems involved (see e.g. [Kimmel et al., 1998], (Swierniak and Smieja, [66])). The authors should discuss this problem and justify the use of four compartmental model (Fig. 4).

Author response: *We first wish to clarify that our model is not a mutation model. We have stated that the transitions are genetic and epigenetic transitions. This includes mutations, insertions, deletions, translocations, copy number variations, and stable epigenetic DNA and histone modifications. The only requirement is that the alteration be stably passed on from parent to daughter cells. We have text in the manuscript and in the legend to Fig.* *4* *(now Fig.* *6* *) to this effect. The variety of transition mechanisms is the reason transition rates were varied over eight orders of magnitude in order to encompass a wide range of mechanisms* [2].

*We thank Dr. Swierniak for the suggestion of comparing our model with the multistage models mentioned in his comments. We have added text in the discussion regarding this issue. In our model, states are defined in terms of drug efficacy. Thus a state in our model may encapsulate multiple molecular configurations, as we mention in the Discussion. In contrast, the multistage models cited by the reviewer intend to capture particular molecular mechanisms -- gene duplication and deletion in their cases. Our model is certainly a simplification and abstraction of the complex mechanisms of molecular alterations. If there is a monotonic relation between quantitative states (e.g., copy number of genes) and drug resistance, and state transitions are relatively homogeneous (e.g., the transition probabilities from 2 to 3 copies and from 3 to 4 copies are in the same scale), then our model is a reasonable approximation. However, when complex relations exist between quantitative states and drug resistance (for instance, drug resistance is maximal at 3 copies and declines with higher and lower copy numbers), or state transitions are highly inhomogeneous (for instance, the transition probabilities from one to two copies is much higher than the transition probabilities from two to three copies), then compartmental models are more adequate to capture the process. Nevertheless, the substantial increase of model complexity in such models will make large-scale simulations intractable.*

Reviewer Comment: 2) Drug resistance is important obstacle against successful chemotherapy but it is not the primary goal of personalized therapy.

Author response: *We agree (as stated by Dr. Swierniak below) that reduction of toxicity is highly desirable. However, based on our experience providing medical care to cancer patients and leading clinical development of numerous targeted agents, we disagree with the contention that minimizing toxicity is the primary goal. Far and away the primary goal in the treatment of a life threatening condition is efficacy. The majority of patients will risk considerable toxicity to optimize survival. Minimizing toxicity is also an important goal, but the primary goal of precision therapy and any therapy for cancer is efficacy. Initial claims of the degree to which precision medicine would also reduce toxicity may have been naïve in our opinion. Most targeted agents attack fundamental pathways common to normal cells, and have significant side effects. Experience has shown that benefit from single targeted agents is short-lived and that targeted programs must knock out numerous related nodes in the intended signalling pathways and redundant pathways in order to have meaningful efficacy. The ability of precision therapy to minimize toxicity under these circumstances may be challenging despite its attempt to exploit differences between patients and between normal and malignant cells*.

Reviewer comment: The author do not discussed how other goals could be reached. For example the problem of side effects and other problems related to chemo-toxicity are incorporated in their simulation study. The only comment on this issue in the manuscript is that some constrains on chemotoxicity are satisfied. The problem of minimization of side effects seems to be one of the most important problems of precise therapy and should be addressed in the paper.

Author response: *Toxicity is a problem that in our opinion needs to be addressed in the context of specific agents, specific toxicities, and specific dosing schedules, not in a generic simulation encompassing all of oncology. In this context, it may be possible to work out strategies in specific cases which minimize toxicity without sacrificing efficacy. However, our paper is focused on patients who suffer from metastatic cancer which is nearly universally fatal untreated, and for the majority of these patients efficacy is the first priority. Dynamics of toxicity are very different, and population dynamic models may not be the best way to evaluate toxicity. Representing toxicity in a generic model is extremely challenging. For example, in a clinical trial of 8000 men with prostate cancer led by one of us, a principal component analysis of safety outcomes identified a 600 dimensional space. Determining which of these safety events were related to therapy and which were due to cancer or comorbidities was a daunting problem even in a randomized placebo controlled trial. Specific dynamic and static constraints representing toxicities can in principle be added in specific cases as needed based on knowledge*.

Reviewer comment: The authors refer to some papers of Gatenby et al. on adaptive therapy but it seems that they have not studied these papers carefully.

Author response: *We have greatly enjoyed reading Gatenby’s papers and have discussed them with him directly at length. Nonetheless, we would be glad to learn additional nuances. This paper was not intended as a review of Gatenby’s work, but we are emphasizing a landmark paper which in our view is still seminal in his thinking. We have modified the text to make it clear that we agree with the adaptive nature of Gatenby’s approach as compared to the current approach of maintaining a constant therapy. However, our approach provides a different way to vary therapy and create an unpredictable, jagged fitness landscape*.

*While we find Gatenby’s ideas intriguing, we differ with him in several important respects. We have seen considerable emphasis on local competition between sub-populations, pitting tumor sub-populations against each other to harness this competition, and relying on inducing evolutionary steady states to stabilize tumor growth utilizing evolutionary game theory and Nash equilibria. In this context, we have seen the recommendation to reduce intensity of therapy against sensitive sub-populations to allow them to compete with resistant sub-populations.*

*In contrast, we view competition as a largely local phenomenon in those lesions approaching their carrying capacity, and one which is magnified in perceived importance by the limited carrying capacity of laboratory systems. We focus on the cells which are clinically relevant for survival of the patient in our opinion, those cells in uncountable metastases of sizes well below the local carrying capacity and only minimally subject to competition. We do not view cancer as an equilibrium process until the end of its course, when the carrying capacity of the whole organism is exceeded, at which point it is too late clinically. Local competition between sub-clones plays little role in the process in our view, when opportunities for metastasis are so readily available. Accordingly, we see intratumoral heterogeneity not as an opportunity for tumor control, but as a threat, providing opportunities for cooperation between tumor cells* [59]*, which has been documented in numerous instances, as well as a reservoir of diversity leading to resistance. Rather than reduce intensity against the sensitive cells in order to allow them to compete with resistant cells, we would focus on finding non-cross resistant therapies to eliminate singly resistant cells before they become multiply resistant. As a by-product, we might coincidentally reduce dose intensity for sensitive cells if all agents cannot be given together in full dose simultaneous combination.*

Reviewer comment: The problem of balance between drug resistance and tumor motility is yet another problem to be discussed.

Author response: *We are not sure which aspect of this complex issue Dr. Swierniak is referring to here. It is important to again emphasize that the patients of interest in this study already likely have widespread undiagnosed micrometastatic disease if not numerous frank metastases. It is therefore too late to prevent metastases. Clearly, there is a relationship between the epithelial-mesenchymal transition required for metastasis and resistance to some therapies; however, this transition reverses upon establishment of a new metastasis* [79]*. Another interesting phenomenon is observed in preclinical models with a limited carrying capacity when a gradient of drug concentrations is established, as is likely to occur in patients due to biophysical properties of tumors which make homogeneous distribution of therapy unlikely* [55, 73]*. In order to avoid local competition in these preclinical model systems, partially resistant cells migrate to the highest drug concentration they can tolerate* [80]*. This phenomenon occurs on a rapid timescale and may contribute to the average short term effectiveness of therapies in some instances. However, its relevance to late relapse, the major phenomenon of interest to this paper, is unclear. Our model lacks spatial resolution, and this may be an interesting future research topic*.

## Additional file

Additional file 1: Long Range Personalized Cancer Treatment Strategies Incorporating Evolutionary Dynamics. (PDF 145 kb)

## Abbreviations

ALTO: Adaptive long-term optimization
ALTO-SMO: Adaptive long-term optimization—serial monotherapy only
CML: Chronic myeloid leukemia
CPU: Central processing unit
DNA: Deoxyribonucleic acid
GB: Gigabytes
GHz: Gigaherz
HIV: Human immunodeficiency virus
HP: Hewlett Packard
IBM: International Business Machines
LLC: Limited Liability Corporation
PNAS: Proceedings of the National Academy of Sciences USA
R_1_: Subclone resistant to drug 1
R_1-2_: Subclone resistant to drugs 1 and 2
R_1-2-3_: Subclone resistant to drugs 1, 2, and 3
R_1-3_: Subclone resistant to drugs 1 and 3
R_2_: Subclone resistant to drug 2
R_2-3_: Subclone resistant to drugs 2 and 3
R_3_: Subclone resistant to drug 3
S: Sensitive subclone
