# Supplementary material for: Long range personalized cancer treatment strategies incorporating evolutionary dynamics
Source: Biol Direct. 2016 Oct 22;11:56. doi: 10.1186/s13062-016-0153-2 (PMC5075220; doi:10.1186/s13062-016-0153-2)
Supplement: Additional file 1: — Long Range Personalized Cancer Treatment Strategies Incorporating Evolutionary Dynamics. (PDF 145 kb) [file 13062_2016_153_MOESM1_ESM.pdf]

Long Range Personalized Cancer Treatment Strategies Incorporating Evolutionary Dynamics, Supplementary Materials

Chen-Hsiang Yeang[1,4], Robert A. Beckman[2,3,4*]

[1]Institute of Statistical Science, Academia Sinica, Taipei, Taiwan.

[2]Department of Oncology, Lombardi Cancer Center, Georgetown University Medical Center, Washington, DC, USA.

[3]Department of Biostatistics, Bioinformatics, and Biomathematics, Lombardi Cancer Center, Georgetown University Medical Center, Washington, DC, USA.

[4]Both authors contributed equally to this work.

[*]Corresponding Author: eniac1915@gmail.com.

Supplementary Results

Comparison between heuristic treatment strategies

Comparison between the five single-step heuristic strategies was previously undertaken [S1]. The absolute results cannot be compared between the two studies. In the previous study we included incurable virtual patients (in whom drugs may only reduce the growth rate of sensitive cells but not produce a net decrease in their numbers), allowed arbitrary dosages for combinatorial treatments subject to a normalized total dosage of 1, and also simulated the two-drug cases only. In contrast, this simulation is restricted to a potentially curable subset (drugs must be capable of producing a net decrease in sensitive cell numbers), allows only fixed dosage combinations (still subject to a normalized dosage of 1), and considers both 2 and 3 drug cases. Nonetheless, certain trends are evident.

The strategies are defined in Table S1. The average performance of treatment outcomes is measured by four quantities: median and mean survival times, fraction of cases with 5-year survival times, and fraction of cured cases (Table S2). The rank orders of average performance derived from the first two measures are consistent: strategy 3 $\approx$ strategy 2.2 $>$ strategy 2.1 $>$ strategy 1 $\gg$ strategy 0 for two-drug cases, and strategy 2.2 $>$ strategy 2.1 $>$ strategy 1 $\approx$ strategy 3 $\gg$ strategy 0 for three-drug cases. The strategy order for two-drug cases is identical to the order previously reported [S1]. As before, the current personalized medicine strategy 0 is considerably worse than other strategies.

Table 1. Treatment strategies. A strategy is a data-driven method for planning a sequence of therapies, based on individual patient data and general oncology knowledge. The strategies discussed in this paper are examples only and not meant to be a comprehensive list. Reproduced from [S1] with permission.

Strategy	Summary	Details
0	Current personalized medicine paradigm.	The patient is treated with the best drug for the observed predominant cell type and switched to the alternative drug upon tumor progression or relapse.
1	Minimize total cell population (i.e. give the drug combination which the model predicts will do so).	Minimized at next 45-day timepoint.
2.1	Minimize the chance of developing incurable R1-2 cells unless radiologically detectable disease burden above a threshold cell number; then minimize total population.	Threshold cell number to be considered radiologically detectable is 10^9 or more. Minimization applies to next 45-day timepoint.
2.2	Same as 2.1 except the threshold value.	Threshold cell number to be considered radiologically detectable is 10^{11} or more.
3	Minimize the total population unless there is an immediate threat of developing an incurable R1-2 cell; then minimize R1-2 cells.	Immediate threat defined as predicted number of R1-2 cells 1 at next 45-day timepoint.
4	Treat the most proximal threat.	Estimated time to mortality from each cell type is compared to estimated time to the first incurable R1-2 cell. The threat estimated to have the shortest time is prioritized in treatment. Mortality is defined as a tumor cell burden of 10^{13}.

Table 2. Comparison of treatment outcomes for 11 strategies. median2: median survival days for two-drug cases. median3: median survival days for three-drug cases. mean2: mean survival days for two-drug cases. mean3: mean survival days for three-drug cases. 5yr2: percentage of two-drug cases with more than 5-year survival time. 5yr3: percentage of three-drug cases with more than 5-year survival time. cure2: percentage of two-drug cured cases. cure3: percentage of three-drug cured cases.

Strategy	Median2	Median3	Mean2	Mean3	5yr2	5yr3	Cure2	Cure3
0	585	720	881.14	1004.72	35.77	29.60	22.99	10.80
single-step 1	810	900	993.24	1170.72	42.46	37.25	26.69	16.37
single-step 2.1	900	945	1014.86	1209.45	44.68	40.25	28.13	18.39
single-step 2.2	1170	1080	1043.59	1234.83	47.26	40.49	30.10	17.61
single-step 3	1215	900	1046.64	1167.03	47.45	35.90	31.92	14.70
multistep 1	990	990	1040.13	1217.35	46.46	39.82	30.14	18.01
multistep 2.1	990	990	1045.17	1234.34	47.24	41.69	30.09	19.34
multistep 2.2	1215	1080	1047.33	1249.62	47.45	41.69	30.12	18.45
multistep 3	1215	990	1048.27	1219.11	47.49	39.30	31.94	16.79
ALTO-SMO	855	1035	1001.36	1226.71	44.73	41.80	34.34	17.86
ALTO	1260	1080	1051.00	1257.00	47.53	43.24	36.76	25.36

Table 3. Numbers of simulation cases where single-step strategies (A) yield clinically superior outcomes compared to corresponding multistep strategies (B) or vice versa. A clinically superior outcome must provide at least a 25% relative improvement and 2 month absolute improvement in survival relative to its comparator strategy. Note that because the 3 drug simulation contains more states and allows doubly resistant cells at time zero, results from it cannot be directly compared to results from the two drug simulation.

# drugs	strategy A	strategy B	A>B	B>A
2	single-step 1	multistep 1	112	28354
3	single-step 1	multistep 1	710	77086
2	single-step 2.1	multistep 2.1	419	16695
3	single-step 2.1	multistep 2.1	14482	39474
2	single-step 2.2	multistep 2.2	13	6199
3	single-step 2.2	multistep 2.2	2	18909
2	single-step 3	multistep 3	0	3748
3	single-step 3	multistep 3	1812	88774

Table 4. Numbers of simulation cases the ALTO strategy is significantly superior or inferior to each indicated strategy. Inferior2: the number of two-drug cases where the ALTO strategy is clinically inferior to each selected strategy. Superior2: the number of two-drug cases where the ALTO strategy is clinically superior to each selected strategy. Inferior3: the number of three-drug cases where the ALTO strategy is clinically inferior to each selected strategy. Superior3: the number of three-drug cases where the ALTO strategy is clinically superior to each selected strategy. A clinically superior outcome must provide at least a 25% relative improvement and 2 month absolute improvement in survival relative to its comparator strategy. Note that because the 3 drug simulation contains more states and allows doubly resistant cells at time zero, results from it cannot be directly compared to results from the two drug simulation.

strategy	Inferior2	Superior2	Inferior3	Superior 3
strategy 0	5	176718	1808	898155
single-step 1	5	32577	3043	171138
single-step 2.1	5	18288	4041	76652
single-step 2.2	5	6378	5631	33093
single-step 3	5	3946	2960	173163
multistep 1	5	6894	3862	79668
multistep 2.1	5	2491	4349	43435
multistep 2.2	5	179	6247	12608
multistep 3	5	197	3888	76074
ALTO-SMO	5	32621	6317	54374

Supplementary Methods

A population dynamic model of tumor growth [S1]

A tumor may consist of multiple subpopulations distinguished by phenotypes of drug resistance. The instantaneous accrual rate of each subpopulation is the intrinsic net growth rate plus the heritable transition rates to it from other subpopulations, minus the drug-induced cell death rate. Given K cell types and D drugs, their population dynamics can be concisely expressed as a vector differential equation:

$$\frac{d\mathbf{X}(t)}{dt} = [(\mathbf{I}+\mathbf{T})g_0 - diag(\mathbf{S}_a\mathbf{d}(t))]U(\mathbf{X}(t)-\mathbf{1})\mathbf{X}(t). \quad (1)$$

where a $K \times 1$ vector $\mathbf{X}(t)$ denotes the size of each subpopulation, g_0 denotes their intrinsic growth rate, $\mathbf{I}$ is a $K \times K$ identity matrix, and $\mathbf{T}$ a $K \times K$ heritable transition rate matrix. A $D \times 1$ vector $\mathbf{d}(t)$ denotes the normalized dosage of each drug where the sum over all drugs equals to one. A $K \times D$ matrix $\mathbf{S}_a$ denotes the sensitivity of each drug on each cell type. $U(\mathbf{X}(t)-\mathbf{1})$ is a component-wise step function. It sets the growth rate to zero when the subpopulation size is below a single cell, preventing exponential growth from a negligible subpopulation.

For D drugs, we consider 2^D cell types manifesting all combinations of resistance phenotypes, where resistance is simplified as being binary (resistant or sensitive) for purposes of simulation. Drug resistance phenotypes are expressed as both binary vectors and alphanumeric symbols. For instance, $(0,0,0)$ or S denotes the cell type sensitive to all three drugs, and $(1,0,0)$ or R_1 denotes the cell type resistant to drug 1 and sensitive to drugs 2 and 3. Cell type $(1,\cdots,1)$ or $R_{1\cdots D}$ is resistant to all drugs and is thus incurable when its population > 1. Quantitatively, in this simulation cell type i is resistant to drug j if $\mathbf{S}_a(i,j) < g_0$ and sensitive otherwise, meaning that the drug has the potential to induce a net negative growth rate or decrease in sensitive cell populations. This defines a clinical situation with highly effective treatment options, which we used for this paper which is examining long term treatment planning. Thus the population in the current study is enriched with patients with a possibility of cure. In Beckman, Schemmann, and Yeang (2012), we also consider cases where the best drugs can only slow the positive net growth rate on sensitive cells, sufficiently to increase expected survival time by 25% in a tumor composed solely of sensitive cells. That definition corresponds to minimal acceptance criteria for oncology drug development in the current state of the art. In Beckman, Schemmann and Yeang (2012) we found that non-standard strategies were also beneficial in situations where available therapies had this lower level of effectiveness. In will be of interest in the future to determine if long range planning is also helpful in this situation. We assume resistance of a drug is caused by a specific set of heritable changes and, in the context of simulations, we assume for simplicity that sensitivities to distinct drugs are independent. For instance, $\mathbf{S}_a(R_1,1) = \mathbf{S}_a(R_{12},1)$.

$\mathbf{T}$ specifies the heritable transition rates between cell types. Heritable transitions may include both sequence mutations and epigenetic alterations on one or multiple genes. We assume in this simulation that resistance of each drug is independently acquired and back transitions from resistant to sensitive phenotypes are negligible, although these assumptions are not required in the more general formulation of the model depicted in the main manuscript. Thus $\mathbf{T}$ can be parameterized by D transition rates of single drug resistance acquisition, denoted by $\delta_1, \cdots, \delta_D$. Only transitions of acquiring one additional drug resistance have nonzero instantaneous rates. For instance, $T(S \to R_1) = T(R_2 \to R_{12}) = \delta_1$, and $T(S \to R_{12}) = 0$. These assumptions are for the convenience of simulation experiments and can be dropped if necessary without affecting the population dynamic model.

A treatment sequence $\mathbf{d}(t)$ is a time-varying function in the simplex $\sum_{i=1}^{D} \mathbf{d}_i(t) = 1, 0 \leq \mathbf{d}_i(t) \leq 1$. We assume the total tumor burden and subpopulations can be estimated at regular intervals, in this case every 45 days. This may be currently possible for hematologic tumors, while promising technologies are emerging for solid tumors (Beckman, Schemmann and Yeang, 2012). Dosages are adjusted according to the surveyed population structure. Hence $\mathbf{d}(t)$ is piecewise constant during the period between two evaluations. In addition, rather than allowing arbitrary dosages we restrict the dosage of a combinatorial therapy to a fixed combination with a total normalized dose of 1. This simulates actual combination therapy, which is generally not given at arbitrary dosages, but only at dosages proven to be safe and tolerable in Phase 1 clinical studies, and generally at reduced dose in combination due to toxicity. Thus, in the simulation there are 3 possible dosage combinations $((1,0),(0,1),(0.5,0.5))$ for two drugs and 7 possible dosage combinations $((1,0,0),(0,1,0),(0,0,1),(0.5,0.5,0),(0,0.5,0.5),(0.5,0,0.5),(\frac{1}{3},\frac{1}{3},\frac{1}{3}))$ for three drugs. The small number of possible dosage combinations makes the simulation experiments with many parameter configurations tractable. In a realistic setting, one or few parameter configurations are inferred from experimental data and the possible dosage combinations may be different as determined by Phase 1 studies. Cure is defined as all subpopulations < 1. Death is defined as occurring when the total number of cells $\geq 10^{13}$.

Heuristic treatment strategies

A treatment strategy is an algorithm for determining the treatment sequence $\mathbf{d}(t)$ according to the surveyed tumor subpopulations and parameters of the population dynamics model. Previously, we considered the current personalized medicine strategy and four treatment strategies that attempted to balance two objectives of reducing tumor size and preventing emergence of incurable multiply-resistant cells (Beckman, Schemmann and Yeang, 2012):

1. Strategy 0: The current personalized medicine strategy. Administer the most effective drug for the largest consensus population and switch to the best alternative drug (as judged by effectiveness against the new largest consensus population) when the tumor relapses or progresses.
2. Strategy 1: Administer the single drug or drug combination that minimizes the predicted total population at the next evaluation point.

3. Strategy 2: Administer the single drug or drug combination that minimizes the emergence risk of an incurable multiply-resistant cell type at the next evaluation point if the total population is below a threshold. Otherwise minimize the predicted total population as in strategy 1. The emergence risk is defined as the total rate of multiply-resistant cell transitions from other cell types. For two-drug cases, $risk = X_{R_1}(t)T(R_1 \rightarrow R_{12}) + X_{R_2}(t)T(R_2 \rightarrow R_{12})$. We chose two population thresholds 10^9 and 10^{11} in simulations and named these algorithms strategies 2.1 and 2.2 respectively.
4. Strategy 3: Administer the single drug or drug combination that minimizes the predicted total population unless the predicted incurable multiply-resistant subpopulation at the next evaluation point ≥ 1. In that case, where there is a threat of emergence of incurable multiple resistance, minimize the predicted incurable multiply-resistant subpopulation.

Multistep extension of heuristic treatment strategies

All the aforementioned strategies are myopic as they propose dosages of the next treatment period only. Treatment sequences that are beneficial in the long run but suffer from short-term losses will be excluded. To improve their outcomes we extend strategies 1-3 to design treatment sequences of multiple periods. Designing a treatment sequence with a fixed number n of look-ahead periods and D non-cross resistant drugs can be viewed as traversing a decision tree illustrated in Supplementary Figure S1. Each node denotes the population structure at the beginning of a treatment period, and the $2^D - 1$ links emanating from this node denote the possible dosage combinations administered during the subsequent period. The root encapsulates the initial population structure, and terminal (leaf) nodes denote the states where either their depths reach the look-ahead period n, the patient is cured (each subpopulation size < 1) or the patient dies (total population size exceeds the mortal threshold 10^{13}). All possible n-step treatment sequences are represented as paths of length n in the decision tree.

Multistep extension of heuristic treatment strategies is realized by a branch-and-bound algorithm on decision trees (Land and Doig, 1960). At the beginning of each of the n treatment periods, a decision tree of subsequent possible n-step treatment sequences is generated. The algorithm traverses all paths along the decision tree and selects the one whose terminal population structure either vanishes (each subpopulation has < 1 cell) or satisfies the criteria stipulated by the heuristic strategy. To reduce unnecessary search a subpath is discarded when the population structure of an intermediate node exceeds the bounds established from previously traversed sequences. The bounds of a node are a list of (total population, multiply-resistant subpopulation) pairs from previously traversed nodes of the same depth. If both numbers of the current node exceed at least one pair from the bound list, then the current subsequence is inferior to some previously traversed subsequences and is thus discarded. These bounds are reasonable approximations since strategies 1-3 concern primarily with total populations and incurable multiply-resistant subpopulations. Population dynamics of the subsequent n periods are then simulated with the selected

treatment sequence. If the patient is neither cured nor dead, then increment n periods (in this simulation) and proceed until the maximum monitoring time (5 years in our simulation study) is reached. In real applications with incomplete information, the strategy would be updated every 45 days based on new experimental measurements. Detailed description of the algorithm is reported below.

An adaptive long-term optimization (ALTO) algorithm for combinatorial treatments

The ultimate goal of cancer treatments is to cure the patients or to maximize their life spans if the former goal is unfeasible. The objectives embedded in strategies 1-3 are reasonable proxies for this goal. Direct maximization of patients' survival durations, however, is difficult. One has to construct the treatment decision tree with a depth equal to patients' life spans or the maximum monitoring time, and find either the longest path or the path leading to cure. With 45 days per treatment period and 5 years' maximum monitoring time, there are $3^{40} = 1.216 \times 10^{19}$ paths for two-drug cases and $7^{40} = 6.367 \times 10^{33}$ paths for three-drug cases. Exhaustive evaluation is clearly intractable.

We propose an approximation algorithm to find an optimal treatment sequence. Two simplification methods are adopted. The first method employs branch-and-bound procedures to eliminate inferior subsequences. Given an initial population structure $\mathbf{X}(0)$, the outcome of a treatment subsequence $\mathbf{d}(t), t \in [0, k\Delta T]$ is dictated by the predicted population structure $\mathbf{X}_{\mathbf{d}(t)}(k\Delta T|\mathbf{X}(0))$ at the end of the subsequence. Thus we can compare two treatment subsequences by examining their predicted population structures. An accurate measure of the outcome prospective for a population structure is the maximum survival time over all possible subsequent treatment sequences. This measure is difficult to calculate but can be bounded from the top and bottom. The maximum survival time is no less than the longest survival time over all valid static treatments (administering an identical dosage combination throughout the whole monitoring duration, and the sum of dosages equals to 1), and no greater than the survival time by administering a full dosage to each drug (unfeasible due to toxicity). In addition, for static treatments ordering relations of survival times are approximately the opposite of the ordering relations of predicted total populations at a far time horizon. These observations are summarized in Proposition 1 below. The proof is stated below.

Proposition 1 Consider two treatment subsequences $\mathbf{d}_1(t)$ and $\mathbf{d}_2(t)$ and denote the predicted population structures following those treatments as $\mathbf{X}_1$ and $\mathbf{X}_2$ respectively. Treat $\mathbf{X}_1$ or $\mathbf{X}_2$ as the initial population, denote $X_1^u(T_{max})$ and $X_2^u(T_{max})$ the minimum total populations at the maximum monitoring time T_{max} over all valid static treatments, and $X_1^l(T_{max})$ and $X_2^l(T_{max})$ the total populations at T_{max} by administering a full dosage of each drug simultaneously (although this is not allowed due to toxicity). The optimal treatment sequences with $\mathbf{X}_1$ and $\mathbf{X}_2$ as initial populations induce trajectories of total populations over time (denote them as $X_1(t)$ and $X_2(t)$ respectively). Suppose the dynamics of total populations in $\mathbf{X}_1$ and $\mathbf{X}_2$ under any static treatment can be approximated by single exponential functions when the total populations are close to or greater than X^{mortal}. Then $\mathbf{d}_2(t)$ yields a longer survival time than $\mathbf{d}_1(t)$ if $X_2^u(T_{max}) < X_1^l(T_{max})$ and $X_1^l(T_{max}) > X^{\text{mortal}}$. Note the assumption concerning the

approximation of population sizes with single exponential functions near the mortality threshold may not always be true, but is true frequently enough to be a useful approximation.

According to Proposition 1, we establish the lower and upper bounds ($X^l(T_{max})$ and $X^u(T_{max})$) of the optimal outcome for each treatment subsequence. A subsequence is discarded if its lower bound $X^l(T_{max})$ lies above the upper bound $X^u(T_{max})$ of some other subsequence.

The second simplification method subdivides the entire treatment durations into n-step episodes and performs branch-and-bound search on the n-step subtrees. Instead of selecting one subsequence as multistep extension of heuristic strategies, we keep multiple subsequences at the end of each episode and continue traversing the following n periods for each subsequence. To avoid a combinatorial explosion we only keep a fixed number of top-ranking sequences according to the geometric means of their lower and upper bounds ($X^l(T_{max})$ and $X^u(T_{max})$). The detailed description of the algorithm is reported in Supplementary Text S1.

To assess whether the treatment benefits stem from dosage combinations of multiple drugs or the advantage of looking multiple steps ahead, we also implement an adaptive long-term optimization algorithm by allowing only the full dosage of a single drug in each time step (adaptive long term optimization: serial monotherapy only, ALTO-SMO). In this variation, the doctor can switch among multiple drugs between treatment periods yet cannot administer multiple drugs concurrently.

Proof of Proposition 1

Proposition 1 is derived from two lemmas.

Lemma 1 The maximum survival time $T^*(\mathbf{X})$ with an initial population structure $\mathbf{X}$ satisfies the following inequalities: $T^u(\mathbf{X}) \leq T^*(\mathbf{X}) \leq T^l(\mathbf{X})$. $T^u(\mathbf{X})$ is the longest survival time over all valid static treatments (administering the same dosage combination throughout the whole monitoring time, and the sum of all drug dosages is one), and $T^l(\mathbf{X})$ is the survival time by administering a full dosage for each drug simultaneously (not really feasible due to toxicity).

Proof $T^u(\mathbf{X}) \leq T^*(\mathbf{X})$ since $T^*(\mathbf{X})$ is no less than the survival time of any valid treatment sequence and static treatments are valid. $T^*(\mathbf{X}) \leq T^l(\mathbf{X})$ since the population following any valid dosage combination is no less than the population following the full dosage of each drug simultaneously. However, $T^l(\mathbf{X})$ is not valid since the full dosages of all drugs together are not allowed due to toxicities.

Lemma 2 Suppose an initial population structure $\mathbf{X}_1$ undergoes an invalid, full-dosage treatment with all drugs simultaneously and another initial population structure $\mathbf{X}_2$ undergoes a valid static treatment. Denote their survival times as T_1 and T_2 and predicted total populations at time T_{max} as $X_1(T_{max})$ and $X_2(T_{max})$. Suppose $X_1(T_{max}) > X^{\text{mortal}}$. Then $T_1 \leq T_2$ if $X_1(T_{\text{max}}) \geq X_2(T_{max})$.

Proof If $X_2(T_{max}) = 0$, then $\mathbf{X}_2$ is cured by the valid static treatment. Thus $T_2 = \infty$ and $T_1 \leq T_2$.

If $min(X_1(T_{\max}), X_2(T_{max})) > 0$, then $\mathbf{X}_1$ is incurable by the full-dosage treatment and $\mathbf{X}_2$ is incurable by the valid static treatment. In either case, the net growth rate matrix $([(\mathbf{I}+\mathbf{T})g_0 - diag(\mathbf{S}_a\mathbf{d}(t))])$ depends only on the fixed dosages of the corresponding treatments and not on initial population structures. The solution of equation 1 has the following form:

$$X_i(t) = \sum_j a_{ij} e^{r_{ij}(t-T_{ij})}. \tag{2}$$

where j denotes the index of distinct net growth rates for subpopulation i (including transitions from other cell types), r_{ij} depends only on the constant net growth rate including transitions from other cell types, and a_{ij} and T_{ij} depend on both the net growth rate and initial populations. $e^{-r_{ij}T_{ij}}$ can be absorbed in the multiplier a_{ij}.

At a far time horizon t, $\sum_i X_i(t)$ is dominated by the term with the highest r_{ij}. Denote the highest r_{ij}'s for the full-dosage treatment in condition 1 and the valid static treatment in condition 2 as r_1 and r_2 respectively. Then at $t = T_{max}$, the total populations can be approximated as $X_1(T) \approx a_1 e^{r_1 t}$ and $X_2(T) \approx a_2 e^{r_2 t}$. Since population 1 receives simultaneous full-dosage treatments and $\mathbf{X}_1$ and $\mathbf{X}_2$ consist of nonzero populations in the same set of cell types, $r_1 \leq r_2$. Moreover, since $X_1(t) \approx a_1 e^{r_1 t}$ and $X_2(t) \approx a_2 e^{r_2 t}$ are monotonic, $X_2(t)$ will eventually overtake $X_1(t)$. $X_1(T_{\max}) \geq X_2(T_{max})$ implies that $T_{max} < T_I$, the intersection time point of $X_1(t)$ and $X_2(t)$. In addition, since $X_1(T_{max}) > X^{\text{mortal}}$, $T_1 \leq T_{max} < T_I$. From the monotonicity of $X_1(t)$ and $X_2(t)$, $T_1 \leq T_2$ if $X_1(T_{\max}) \geq X_2(T_{max})$.

Proof of Proposition 1

The relations between the prediction total populations and survival times of two initial populations under three treatment schemes are shown in Supplementary Figure S1. From lemma 2, if $X_2^u(T_{max}) < X_1^l(T_{max})$, then, subjected to the assumptions above, survival times $T_2^u > T_1^l$. From lemma 1, $T_1^u \leq T_1^* \leq T_1^l$ and $T_2^u \leq T_2^* \leq T_2^l$. Thus $T_2^* > T_1^*$.

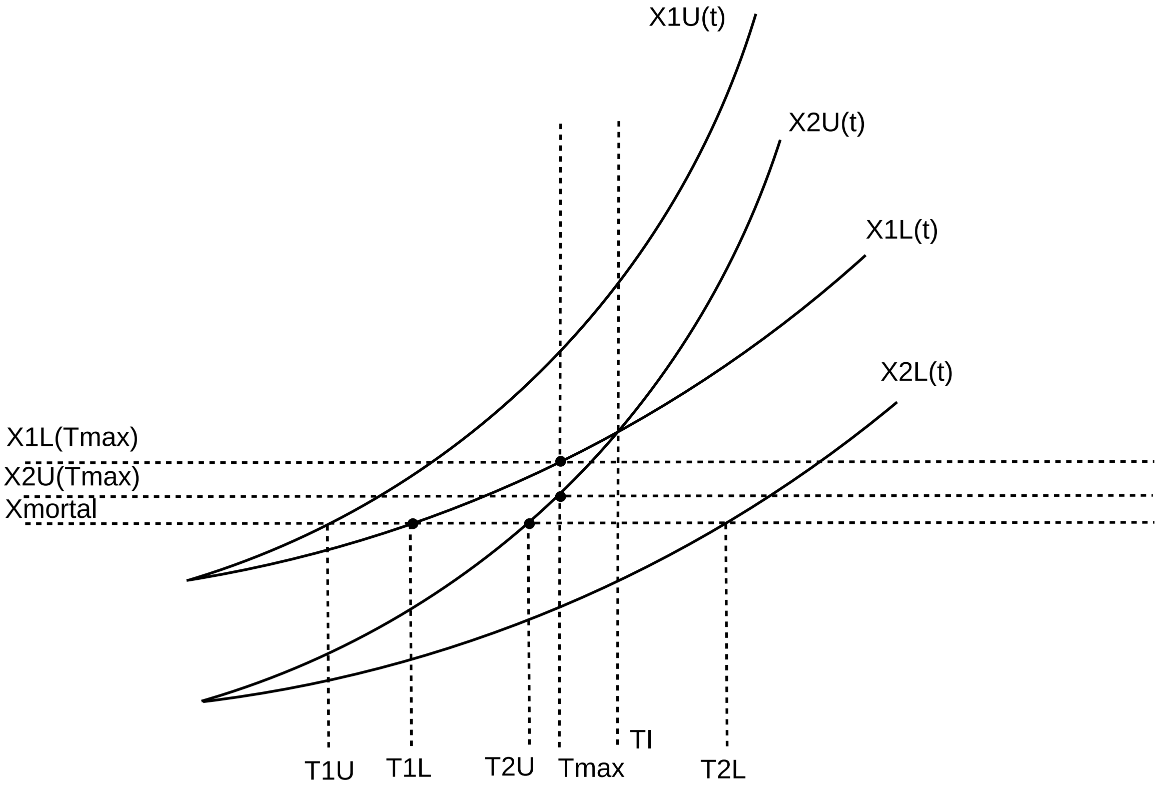

Fig. 1. Relations between the predicted total populations and survival times of two initial populations. $X_1^l(t)$ is the total population response by administering the full-dosage treatment. $X_1^u(t)$ is the total population response by administering the best valid static treatment. $X_2^u(t)$ and $X_2^l(t)$ are analogously defined. If $X_1(T_{\max}) > X_2(T_{max})$, then $T_2^u > T_1^l$ under the aforementioned conditions. Since $T_1^u \leq T_1^* \leq T_1^l$ and $T_2^u \leq T_2^* \leq T_2^l$ (lemma 1), $T_2^u > T_1^l$ implies $T_2^* > T_1^*$.

Parameter names and values in population dynamics models

Table 5. Parameter names and values in population dynamics models. See [S1] for additional explanation of parameter definitions and values.

name	two-drug values	three-drug values
g_0	$0.001, 0.0026, 0.007, 0.0184, 0.0487, 0.1287, 0.34$	$0.001, 0.0074, 0.055$
$\frac{x_S(0)}{x(0)}$	-	$0.1, 0.9$
$\frac{x_{R_1}(0)}{x(0)}$	$0, 10^{-9}, 10^{-7}, 10^{-5}, 10^{-3}, 0.1, 0.9$	$0, 10^{-5}, 10^{-3}, 0.1, 0.9$
$\frac{x_{R_2}(0)}{x(0)}$	$0, 10^{-9}, 10^{-7}, 10^{-5}, 10^{-3}, 0.1, 0.9$	$0, 10^{-5}, 10^{-3}, 0.1, 0.9$
$\frac{x_{R_3}(0)}{x(0)}$	-	$0, 10^{-5}, 10^{-3}, 0.1, 0.9$
$\frac{x_{R_{12}}(0)}{x(0)}$	-	$0, 10^{-5}, 10^{-3}$
$\frac{x_{R_{23}}(0)}{x(0)}$	-	$0, 10^{-5}, 10^{-3}$
$\frac{x_{R_{13}}(0)}{x(0)}$	-	$0, 10^{-5}, 10^{-3}$
$\frac{S_a(S,D_1)}{g_0}$	$5.6 \times 10^{-4}, 0.0054, 0.0517, 0.4964, 4.7683, 45.8045, 440$	$0.5, 5$
$\frac{S_a(S,D_2)}{S_a(S,D_1)}$	$4 \times 10^{-4}, 0.0015, 0.0054, 0.02, 0.0737, 0.2714, 1$	$0.1, 0.3, 1$
$\frac{S_a(S,D_3)}{S_a(S,D_1)}$	-	$0.1, 0.3, 1$
$\frac{S_a(R_1,D_1)}{S_a(S,D_1)}$	$0, 10^{-5}, 9.5635 \times 10^{-5}, 9.1461 \times 10^{-4}, 0.0087, 0.0837, 0.8$	$0, 0.3, 1$
$\frac{S_a(R_2,D_2)}{S_a(S,D_2)}$	$0, 10^{-5}, 9.5635 \times 10^{-5}, 9.1461 \times 10^{-4}, 0.0087, 0.0837, 0.8$	$0, 0.3, 1$
$\frac{S_a(R_3,D_3)}{S_a(S,D_3)}$	-	$0, 0.3, 1$
$T(S \to R_1)$	$10^{-11}, 2.154 \times 10^{-10}, 4.642 \times 10^{-9}, 10^{-7}, 2.154 \times 10^{-6}, 4.642 \times 10^{-5}, 10^{-3}$	$10^{-11}, 10^{-9}, 10^{-7}, 10^{-5}, 10^{-3}$
$T(S \to R_2)$	$10^{-11}, 2.154 \times 10^{-10}, 4.642 \times 10^{-9}, 10^{-7}, 2.154 \times 10^{-6}, 4.642 \times 10^{-5}, 10^{-3}$	$10^{-11}, 10^{-9}, 10^{-7}, 10^{-5}, 10^{-3}$
$T(S \to R_3)$	-	$10^{-11}, 10^{-9}, 10^{-7}, 10^{-5}, 10^{-3}$

Simulation setup

We simulated equation 1 of the main text for two-drug and three-drug cases. The two-drug model consists of 9 free parameters listed on Supplementary Table 1, including the intrinsic growth rate, initial subpopulations, drug sensitivity ratios and heritable transition rates. The initial total population is fixed to 5×10^9 and the initial R_{12} population is zero. Each parameter can take 7 possible values. The ranges of values were chosen to encompass the entire range of likely values over solid and liquid tumors based on experimental and clinical data, thus providing a broad survey of initial conditions likely to be encountered clinically. The $7^9 = 40353607$ parameter configurations were filtered with the following criteria:

1. Sensitive cell types can be eradicated by each drug ($S_a(S,1) > g_0, S_a(S,2) > g_0$). Thus in contrast Beckman,Schemman, and Yeang (2012), we consider only fundamentally curable cases.
2. Multiply-resistant cell types cannot be eradicated by any drug ($S_a(R_{12},1) < g_0, S_a(R_{12},2) < g_0$).
3. The patient can be cured by simultaneous full dosages of all drugs (an invalid option due to toxicity) but cannot be cured by any valid static treatment.

These criteria rule out the cases where the patient is cured or dies regardless of treatment strategies employed. 764104 parameter configurations pass the filters.

The three-drug model consists of 17 free parameters listed on Supplementary Table 1. The number of possible values for each parameter varies in order to reduce the total number of parameter configurations. The 364500000 parameter configurations were filtered with the following two criteria in addition to the aforementioned three criteria:

1. The potency of drugs on sensitive cells follows: drug 1 $\geq$ drug 2 $\geq$ drug 3. This is simply an arbitrary drug labeling convention and does not reduce the information content of the simulation.
2. The initial size of a doubly-resistant subpopulation is no greater than the initial sizes of two singly-resistant subpopulations from which it is derived. This condition is not necessary for the model, but was done to reduce the computational burden.

1723116 parameter configurations pass these filters.

For each parameter configuration, representing a virtual patient, we implemented 11 treatment strategies, simulated their population dynamics, and calculated survival times under those regimens. Treatment strategies include the aforementioned five heuristics, four multistep extensions (strategy 0 excluded), and the ALTO algorithm for all valid dosage combinations and for mono therapies alone (ALTO-SMO). The following parameters were fixed throughout simulations: the length of constant treatment periods – 45 days, the maximum monitoring/treatment duration – 1800 days, the number of look-ahead steps for multistep extension – 5 steps, the depth of subtrees in the ALTO algorithm – 5. The maximum number of tracked subsequences in the ALTO algorithm was 500 for two-drug cases and 50 for three-drug cases. The survival time of a patient cured before 1800 days was reported as 1845 days. This will tend to underestimate the benefit of increased cure rate when expressed as mean or median survival. Simulations were undertaken on 23 HP DL360 G7 servers in parallel. Each server contained dual Intel(R) Xeon(R) CPUs E5520 with 2.27 GHz and 24 GB main memory. The total running time was 20 hours for two-drug cases and 10 days for three-drug cases.

Description of dynamic programming treatment design algorithms

Multistep extension of the heuristic strategies **Inputs:** Model parameters π in equation 1 of the main text, initial subpopulation sizes, fixed number of look-ahead steps n ($n = 5$ in simulation), treatment period ΔT (45 days in simulation).

Outputs: A treatment sequence $\mathbf{d}(t), t \in [0, T]$, where T is either the maximum monitoring time (typically 5 years) or the time to mortality (total population $\geq 10^{13}$) or complete cure (total population < 1).

Procedures:

1. Set $t = 0, \mathbf{X}(t) = \text{initial population}, \mathbf{d}(t) = []$.

2. While $t \leq T$ and the patient is neither dead nor cured,
   (a) Generate all n-step candidate treatment sequences by running the program $recurse\_branch\_bound(\mathbf{X}(t), \pi, \mathbf{d}(t), 0)$.
   (b) Select the treatment sequence $\hat{\mathbf{d}} = (\hat{\mathbf{d}}(0), \cdots, \hat{\mathbf{d}}(n-1))$ that optimizes the criteria of strategy 1, 2 or 3.
   (c) Simulate population dynamics by running the program $(\mathbf{X}(t+\Delta T), \cdots, \mathbf{X}(t+n\Delta T)) = simulate(\mathbf{X}(t), \pi, \hat{\mathbf{d}})$.
   (d) Augment $\hat{\mathbf{d}}$ to $\mathbf{d}(t)$.
   (e) Assign $\mathbf{X}(t) \leftarrow \mathbf{X}(t + n\Delta T)$.
   (f) $t \leftarrow t + n\Delta T$.
3. Return $\mathbf{d}(t)$.

$recurse\_branch\_bound(\mathbf{X}(t), \pi, \mathbf{d}(t), \text{depth})$:

1. Terminate if depth $= n$.
2. Terminate if the patient is dead or cured.
3. Terminate if the total and multiply-resistant populations both exceed those of some other nodes of the same depth.
4. For each possible dosage combination $\hat{\mathbf{d}}$,
   (a) Run the simulation program in one step $\mathbf{X}(t + \Delta T) = simulate(\mathbf{X}(t), \pi, \hat{\mathbf{d}})$
   (b) Augment $\hat{\mathbf{d}}$ to $\mathbf{d}(t)$, depth $\leftarrow$ depth $+ 1$.
   (c) Run the recursive function $recurse\_branch\_bound(\mathbf{X}(t), \pi, \mathbf{d}(t), \text{depth})$

$simulate(\mathbf{X}(t), \pi, \hat{\mathbf{d}})$: Analytically solve equation 1 of the main text. See [S1].

An adaptive long term optimization (ALTO) algorithm for combinatorial treatments **Inputs:** Model parameters π in equation 1 of the main text, initial subpopulation sizes, fixed number of look-ahead steps n ($n = 5$ in simulation), treatment period ΔT (45 days in simulation), the maximum number N_c of candidate treatment sequences considered.

Outputs: A treatment sequence $\mathbf{d}(t), t \in [0, T]$, where T is either the maximum monitoring time (typically 5 years) or the time to mortality (total population $\geq 10^{13}$) or complete cure (total population < 1).

Procedures:

1. Set $k = 0, \mathbf{X}(t) =$ initial population, global candidate treatment sequences=$\{\}$.
2. While $k\Delta T \leq T$ and the patient is neither dead nor cured,
   (a) For each k-step global candidate sequence $\mathbf{d}(t)$, generate all n-step valid subsequences by running the program $recurse\_branch\_bound(\mathbf{X}(t), \pi, \mathbf{d}(t), 0)$.

(b) For each $(k+n)$-step treatment sequence, calculate the predicted total population at time T with the following constant dosages throughout the whole episode: (1)full dosage of each drug $((1, \cdots, 1))$, (2)the valid dosage that yields the lowest total population. The predicted total populations x_l and x_u of those two schemes serve as lower and upper bounds of treatment outcomes.

(c) Remove the $(k+n)$-step sequences whose lower bounds exceed the upper bounds of some other sequences.

(d) If the total number of remaining sequences exceeds N_c, then sort them according to the geometric means of their x_l and x_u values. Keep the top N_c sequences and remove others.

(e) Assign the remaining $(k+n)$-step sequences to the global candidate treatment sequences.

(f) $k \leftarrow k+n$.

3. Return an arbitrary member from the global candidate treatment sequences.

recurse_branch_bound$(\mathbf{X}(t), \pi, \mathbf{d}(t), \text{depth})$: see above.

simulate$(\mathbf{X}(t), \pi, \hat{\mathbf{d}})$: see above.

References

[S1] Beckman R.A., Schemmann G.S., and Yeang C.H., (2012). **Impact of genetic dynamics and single-cell heterogeneity on development of nonstandard personalized medicine strategies for cancer.** *Proc. Natl. Acad. Sci. USA*, **109(36)**, 14586-14591.

[S2] Land A.H. and Doig A.G., (1960). **An automatic method of solving discrete programming problems.** *Econometrica*, **28(3)**, 497-520.
